# Cardiac complications in patients with COVID-19: a systematic review

**DOI:** 10.1186/s44158-022-00046-7

**Published:** 2022-04-27

**Authors:** E. Brogi, F. Marino, P. Bertini, G. Tavazzi, F. Corradi, F. Forfori

**Affiliations:** 1 Department of Anesthesia and Intensive Care, Azienda Ospedaliero Universitaria Pisana, Via Paradisa 2, 56124 Pisa, Italy; 2 Department of Surgical, Medical, Molecular Pathology and Critical Care Medicine, University of Pisa, Pisa, Italy; 3 Department of Emergency-Covid Intensive Care, Azienda Ospedaliera Universitaria di Bologna - Policlinico “S. Orsola Malpighi”, Bologna, Italy; 4 Department of Anesthesia and Critical Care Medicine, Azienda Ospedaliero Universitaria Pisana, Via Roma n° 67, 56126 Pisa, Italy; 5 Department of Clinical-Surgical, Diagnostic and pediatric Sciences, University of Pavia, Via Alessandro Brambilla, 74, 27100 Pavia, Italy; 6 Anesthesia and Intensive Care, Fondazione Policlinico San Matteo IRCCS, Viale Camillo Golgi, 19, 27100 Pavia, PV Italy; 7 Department of Anesthesia and Intensive Care, Ente Ospedaliero Ospedali Galliera, Genova, Italy

**Keywords:** Acute myocardial injury, Acute myocardial infarction, Acute cardiac injury, Cardiac complications, Heart failure, Arrhythmia, Mortality, Coronavirus, Coronavirus infection

## Abstract

Cardiac complications in patients with COVID-19 have been described in the literature with an important impact on outcome. The primary objective of our systematic review was to describe the kind of cardiac complications observed in COVID-19 patients and to identify potential predictors of cardiovascular events. The secondary aim was to analyze the effect of cardiac complications on outcome.

We performed this systematic review according to PRISMA guidelines using several databases for studies evaluating the type of cardiac complications and risk factors in COVID-19 patients. We also calculated the risk ratio (RR) and 95% CI. A random-effects model was applied to analyze the data. The heterogeneity of the retrieved trials was evaluated through the *I*^2^ statistic. Our systematic review included 49 studies. Acute cardiac injury was evaluated in 20 articles. Heart failure and cardiogenic shock were reported in 10 articles. Myocardial infarction was evaluated in seven of the papers retrieved. Takotsubo, myocarditis, and pericardial effusion were reported in six, twelve, and five articles, respectively. Arrhythmic complications were evaluated in thirteen studies. Right ventricular dysfunction was evaluated in six articles. We included 7 studies investigating 2115 patients in the meta-analysis. The RR was 0.20 (95% CI: 0.17 to 0.24; *P* < 0.00001, *I*^2^ = 0.75). Acute cardiac injury represented the prevalent cardiac complications observed in COVID-19 patients (from 20 to 45% of the patients). Patients with acute cardiac injury seemed to be significantly older, with comorbidities, more likely to develop complications, and with higher mortality rates. Acute cardiac injury was found to be an independent risk factor for severe forms of SARS-CoV-2 infection and an independent predictor of mortality. Due to the scarce evidence, it was not possible to draw any conclusion regarding Takotsubo, myocarditis, pleural effusion, and right ventricular dysfunction in COVID-19 patients. Noteworthy, possible arrhythmic alterations (incidence rate of arrhythmia from 3 to 60%) in COVID-19 patients have to be taken into account for the possible complications and the consequent hemodynamic instabilities. Hypertension seemed to represent the most common comorbidities in COVID-19 patients (from 30 to 59.8%). The prevalence of cardiovascular disease (CVD) was high in this group of patients (up to 57%), with coronary artery disease in around 10% of the cases. In the majority of the studies retrieved, patients with CVD had a higher prevalence of severe form, ICU admission, and higher mortality rates.

## Background

From December 2019, when a new severe respiratory disease was reported in Wuhan, China, the infection caused by a novel coronavirus quickly spread across the globe causing a pandemic with devastating consequences [1] and challenging health care organization [2].

Although COVID-19 is characterized mainly by upper and lower respiratory system involvement, with consequent respiratory symptoms at different grades of severity (from cough to shortness of breath, to dyspnea, to respiratory failure), evidence of extrapulmonary manifestations is established [3–5] including venous and arterial thrombotic manifestations [6, 7]. Cardiac complications (i.e., acute cardiac injury, cardiogenic shock, heart failure, arrhythmia, right ventricular dysfunction) have been described in the literature with an important impact on outcome [8]. Several mechanisms are implicated in the genesis of cardiac complications in patients with COVID-19: direct myocardial cells injury (through ACE2 receptors), systemic inflammation, catecholamine surge, cytokine storm, electrolyte imbalance, and hypoxia [9]. Consequently, it was extremely important to further report the cardiological events related to COVID-19 and identify possible risk factors (e.g., hypertension, diabetes) in order to stratify patients with increased risk of cardiovascular events during SARS-CoV-2.

The primary objective of our systematic review was to describe the type of cardiac complications in patients with COVID-19 and the potential associated risk factors (i.e., hypertension, diabetes, previous stroke, previous cardiovascular events, respiratory diseases, COPD, malignancy, chronic kidney disease, chronic liver disease) that may allow the identification of patients more exposed to cardiovascular events represented our secondary aim. The secondary aim was to analyze the relation between cardiac complications and outcome.

## Methods

### Protocol and registration

The authors performed this systematic review following the Preferred Reporting Items for Systematic Reviews and Meta-Analyses (PRISMA) statements [10].

PROSPERO registration number: CRD42021231922.

### Search

We systematically explored the US National Library of Medicine database (MEDLINE), Web of Science, Google Scholar, Scopus, Cinhal Database, and Cochrane Central Register of Controlled clinical studies (CENTRAL) for RCT, case report, case series, letters to the editor, correspondence, abstracts, and brief reports from 2019 to 2021. We did not impose any restrictions for language. The search criteria were (arrhythmia OR cardiac ischemia OR cardiogenic shock OR acute myocardial infarction OR Heart failure OR Myocarditis OR right ventricle dysfunction) AND (COVID-19 OR coronavirus OR coronavirus pandemic OR coronavirus pneumonia) AND (comorbidities OR hypertension OR Diabetes OR dyslipidemia OR smoker OR chronic obstructive pulmonary disease OR COPD OR chronic kidney disease OR chronic kidney failure OR stroke OR Chronic liver failure OR hyperthyroidism OR dementia). Furthermore, we hand-searched the reference lists of the articles in order to find all relevant articles missed by electronic search. The research was limited to randomized, observational, and retrospective studies, case report, case series, letters to the editor, correspondence, abstracts, and brief reports on human subjects that describe the type of cardiac complications in COVID patients and the possible clinical factors that may allow the identification of patients with a high risk of cardiovascular events.

### Eligibility and study selection

We included randomized, case-controlled, and cohort studies (both prospective and retrospective), case report, and case series evaluating the type of cardiac complications in COVID-19 patients and the possible clinical risk factors. Reviews, conference proceedings, meta-analyses, and international expert recommendations were not included. The authors screened all search results, titles, and abstracts of retrieved articles to assess eligibility and then obtained a full-length manuscript for all the included studies. Among the publications identified, we excluded studies of irrelevance to the topic, technical descriptions, proceeding, and non-human model studies.

### Data collection

A data collection form was created with the following main study characteristics: authors, year of publication, country, number of patients included, study design, primary outcome, other outcomes, number of treatment/control, inclusion criteria, exclusion criteria, quality of the evidence, age, sex, cardiological comorbidities, other comorbidities, COVID-19 severity, characteristics of cardiac complication analyzed, other cardiological complications (respiratory, neurological, infections), and outcomes. The authors screened all search results by literature search. One investigator (F.M.) was in charge of collecting the data and assessing the methodological validity of all the eligible studies. Then, all the data were verified by two further investigators (E.B., F.F.).

### Outcome

The primary objective was to describe the type of cardiac complications observed in COVID-19 patients and to identify possible clinical risk factors (i.e., hypertension, diabetes, dyslipidemia, smoking, coronary heart disease previous stroke, previous cardiovascular events, respiratory diseases, COPD, malignancy, chronic kidney disease, chronic liver disease) that may allow the identification of patients with a high risk of cardiovascular complications in comparison with patients without risk factors. The secondary aim was to analyze the effects of cardiac complications on mortality.

### Assessment of study quality

For randomized studies, the selected articles were evaluated using the 3-item, 5-point Oxford Quality Scale. Studies are rated on the basis of three methodological features: 2 points for descriptions of randomization, 2 points for the description of the blinding process, and 1 point for the description of withdrawal. We excluded from this review all articles which did not obtain a minimum score of 2. The Newcastle-Ottawa Scale was used for case-controlled or cohort studies quality assessment. Each study is judged on three broad perspectives: the choice of the study groups, the comparability of the groups, and also the ascertainment of either the exposure or the outcome of interest for case-control or cohort studies, respectively.

### Strategy for data synthesis

**Fig. 1:**
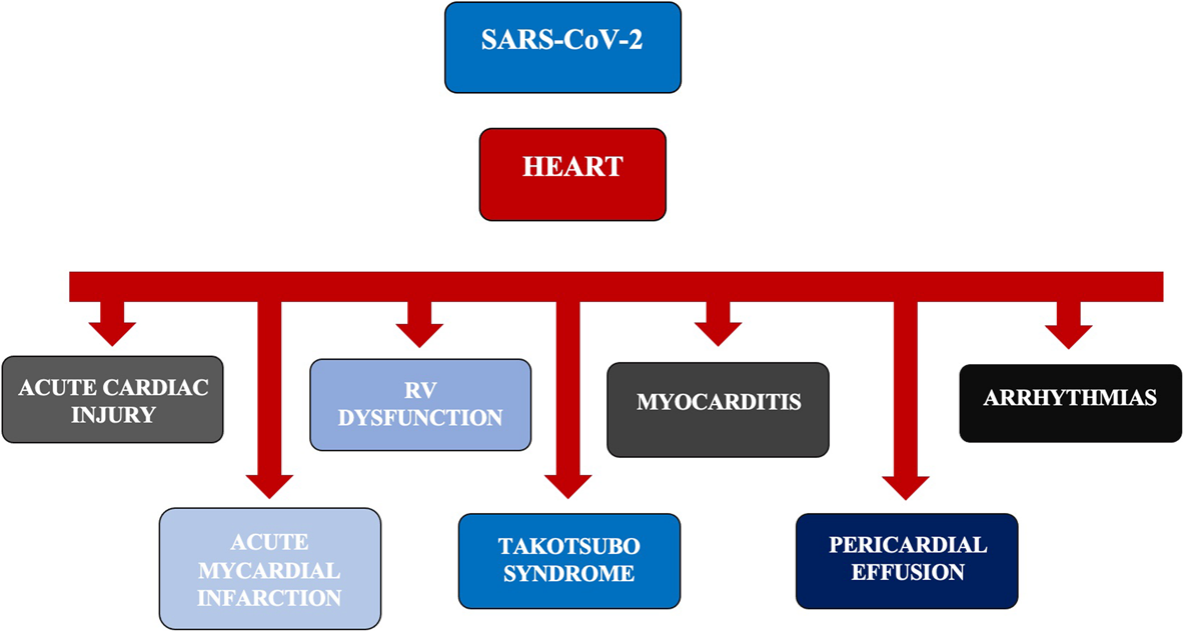
Graphical representation of cardiac complications in SARS- CoV-2 patients

We created a descriptive summary table with the main characteristics of included studies (Table 1). Then, we also created descriptive tables for the following cardiac complications (from Tables 2, 3, 4, 5, and 6; a graphical representation of the cardiac complications is shown in Fig. 1):

1. Acute cardiac injury
2. Acute myocardial infarction
3. Takotsubo syndrome
4. Myocarditis
5. Pericardial effusion
6. Arrhythmias
7. Right ventricular dysfunction

**Table 1:** Characteristics and outcomes of retrieved trials

Authors	Month/year of publication	Country	Study design	Primary outcome	Other outcomes	All patients	No. of treatment/control	Inclusion criteria	Exclusion criteria	Quality of evidences
Amaratunga et al. [11]	May 2020	USA	Case series	To report sinus bradycardia as a potential manifestation of COVID-19	-Association between heart rate and temperature/blood pressure/oxygen saturation -Association between heart rate and medication	4	NA	NA	NA	NA
Arentz et al. [12]	March 2020	USA	Case series	To describe the clinical presentation, characteristics, and outcomes of patients COVID-19 admitted in Evergreen Hospital’s ICU	NA	21	NA	NA	NA	NA
Bangalore et al. [13]	April 2020	USA	Case series	To describe clinical experience	Electrocardiographic evidence	18	8/10	Patients with STEMI	NA	NA
Barton et al. [14]	June 2020	USA	Case series: autopsy	To share observations on COVID-19, based on 2 autopsies	To share experience on precautions and equipment to perform autopsies	2	NA	-Patients not treated for COVID-19 -Not tested patients before death	NA	NA
Cao et al. [15]	March 2020	China	Retrospective	To describe therapies, clinical and laboratory characteristics, and short-term outcomes of COVID-19 patients admitted in ICU	NA	102	18–84	Patients with COVID-19 hospitalized in Wuhan University Zhongnan Hospital between January 3 and February 1, 2020	NA	7^#^
Chen et al. [16]	May 2020	China	Retrospective	To identify how COVID-19 damages the cardiovascular system, at the level myocardial, conduction system, heart function, and blood pressure, especially in severe cases	To highlight potential risk factors for the severity of COVID-19	54	NA	Severe and critical clinical cases, hospitalized in Zhongshan hospital between January 27 and February 28, 2020	NA	7^#^
Chen et al. [17]	May 2020	China	Retrospective	Comparing the demographic, clinical, laboratory, and radiological characteristics of patients with different clinical outcomes	NA	274	113–161	Patients, admitted to the Tonji Hospital between January 13 and February 12, 2020, who died or discharged until February 28, 2020	NA	7^#^
Dabbagh et al. [18]	April 2020	USA	Case report	Description of a clinical case	NA	1	NA	NA	NA	NA
Deng et al. [19]	April 2020	China	Retrospective	To investigate COVID-19 patients’ clinic and therapy, focusing on heart condition	To provide information about possible myocarditis or myocardial injury caused by COVID-19	112	45/67	Patients admitted between January 6 and February 20, 2020, diagnosed with COVID-19 at Renmin Hospital in Wuhan	NA	7^#^
Dominguez-Erquicia et al. [20]	May 2020	Spain	Case Report	Report	NA	1	NA	NA	NA	NA
Doyen et al. [21]	April 2020	France	Clinical case	Report	NA	1	NA	NA	NA	NA
Du et al. [22]	April 2020	China	Retrospective	Retrospective	Describe clinical features	85	NA	All consecutive patients with severe COVID-19 admitted to Hannan Hospital and Wuhan Union Hospital, between January 9 and February 15, 2020	NA	7^#^
Du et al. [23]	April 2020	China	Multicenter observational study	To describe hospitalization and critical care of patients who died of COVID-19 pneumonia	NA	109	51/58	NA	NA	7^#^
Dweck et al. [24].	September 2020	UK	International survey	To improve knowledge about cardiac manifestations of COVID-19	To find patients who would benefit most from echocardiography	1216	667/549	Patients with confirmed or a high probability of COVID-19 with collected transthoracic echocardiogram in hospital setting from April 3 to April 20, 2020	Not available data	7^#^
Guo et al. [25]	July 2020	China	Retrospective	To investigate the association between cardiovascular disease and myocardial injury with fatal outcomes	NA	187	135/52	Patients with a diagnosis of COVID-19, according to the guidance of WHO, admitted to the Seventh Hospital of Wuhan City, China, from January 23 to February 23, 2020	Incomplete data	7^#^
Hu et al. [26]	March 2020	China	Case report	Helpful in treating similar patients	NA	1	NA	NA	NA	NA
Huang et al. [27]	January 2020	China	Report of data	To describe epidemiological, clinical, laboratory, and radiological characteristics, treatment, and outcomes of patients confirmed with COVID-19	To compare ICU and non-ICU patients	41	13/28	Patients with laboratory-confirmed COVID-19 infection by RT-PCR and next-generation sequencing	NA	7^#^
Hussain et al. [28]	June 2020	USA	Case report	Report	NA	1	NA	NA	NA	NA
Inciardi et al. [29]	March 2020	Italy	Case report	To describe the presentation of acute myocarditis in patients with COVID-19	NA	1	NA	NA	NA	NA
Kim HN et al. [30]	June 2020	Korea	Case report	To present their first case of PCI for a patient with COVID-19	NA	1	NA	NA	NA	NA
Kim IC et al. [31]	April 2020	Korea	Case report	Report	NA	1	NA	NA	NA	NA
Kunal et al. [32]	November 2020	India	Retrospective	To evaluate the cardiovascular complications and their impact on outcomes	NA	108	28/80	Symptomatic laboratory-confirmed COVID-19	Asymptomatic patients No cardiac documentation	7^#^
Kuno et al. [33]	May 2020	USA	Retrospective	To investigate the relation between cardiovascular disease and mechanical ventilation and mortality	NA	8438	NA	COVID-19 patients	NA	6^#^
Meyer et al. [34]	April 2020	Switzerland	Case report	Description of Takotsubo case	NA	1	NA	NA	NA	NA
Monmeneu et al. [35]	May 2020	Spain	Case report	To provide evidence obtained with CMR of extensive myocardial involvement secondary to SARS-CoV-2 infection	NA	1	NA	NA	NA	NA
Pascariello et al. [36]	June 2020	Italy	Case report	To describe a case of cardiogenic shock due to COVID-19-related myocarditis	NA	1	NA	NA	NA	NA
Paul et al. [37]	July 2020	France	Case report	Report	NA	1	NA	NA	NA	NA
Peigh et al. [38]	April 2020	USA	Case series	To describe clinical characteristics, potential mechanisms, and outcomes of COVID-19 patients who develop de novo sinus node dysfunction	NA	2	NA	NA	NA	NA
Ruan et al. [39]	March 2020	China	Retrospective	To identify clinical predictors of mild and severe patient outcome	NA	150	68/82	NA	NA	6^#^
Sala et al. [40]	April 202	Italy	Case report	Report	NA	1	NA	NA	NA	NA
Seecheran et al. [41]	April 2020	Trinidad and Tobago	Case report	Description	NA	1	NA	NA	NA	NA
Shao et al. [42]	May 2020	China	Retrospective	To describe COVID-19 progression and risk profiles for mortality in non-survivors	NA	18	NA	Patients died from 155 patients who met the criteria of COVID-19 clinical diagnosis based on the National Health Commission of China Guidelines (7th edition)	Patients discharged	7^#^
Shi et al. [43]	July 2020	China	Retrospective cohort	To examine the association between cardiac injury and mortality	NA	416	82/334	Consecutive patients admitted to Renmin Hospital with confirmed COVID-19, according to WHP interim guidance	No record of cardiac markers	7^#^
Shi et al. [44]	May 2020	China	Retrospective	To determinate the predictive value of myocardial injury markers on hospital death	To investigate features and potential causes of myocardial injury in severe COVID-19 patients	671	106/565	All patients admitted to Renmin Hospital of Wuhan University with confirmed severe COVID-19	Cases with mild COVID-19, duplicated cases, and cases without available medical information	7^#^
Solano-Lopez et al. [45]	May 2020	Spain	Case report	To describe a case of inverted Takotsubo	NA	1	NA	NA	NA	NA
Stefanini et al. [46]	June 2020	Italy	Retrospective	To evaluate the incidence, clinical presentation, angiography, and outcomes of STEMI in patients with COVID-19	NA	28	NA	All patients with confirmed COVID-19 who underwent an urgent angiography because of STEMI between February 20 and March 30, 2020	NA	6^#^
Szekely et al. [47]	July 2020	Israel	Prospective	Comprehensive echocardiographic evaluation—frequency of cardiac abnormalities	Echocardiographic parameters stratified by troponin levels and clinical condition	100	NA	Consecutive patients hospitalized with COVID-19 patients	Echocardiographic evaluation not performed	6^#^
Stӧbe et al.	August 2020	Germany	Observational study	To characterize the myocardial effects by echocardiography	NA	18	NA	Patients with SARS-CoV-2 admitted at Leipzing University Hospital and at Community Hospital Halle (Saale) in April 2020	NA	7^#^
Tavazzi et al. [48]	April 2020	Italy	Case report	To describe a case of myocardial biopsy to prove SARS-CoV-2 localization in myocardium	NA	1	NA	NA	NA	NA
Villanueva et al. [49]	June 2020	USA	Case report	To describe heart failure and arrhythmia as possible symptoms of COVID-19	NA	1	NA	NA	NA	NA
Wan et al. [50]	July 2020	China	Retrospective	To describe the epidemiological and clinical features, laboratory and radiology findings, and outcomes of COVID-19 patients	NA	135	95/40	All patients with confirmed COVID-19 admitted to the Chongqing University Three Gorges Hospital from January 23 to February 8, 2020	NA	NA
Xie et al. [51]	August 2020	China	Retrospective	To evaluate the effects of cardiovascular disease on COVID-19	To evaluate laboratory and clinical characteristics of COVID-19 patients	62	38/24	All patients with confirmed COVID-19 admitted to the Hospital from February 15 to March 14, 2020	Suspected diagnosis	6^#^
Xiong et al. [52]	October 2020	China	Retrospective	To provide a recent description of clinical features of patients	To provide assistance in managing cardiovascular disorders in COVID-19 patients	116	61/55	Laboratory-confirmed COVID-19 admitted to the General Hospital of Central Theater Command (Wuhan, China) from January 20 to March 8, 2020	NA	7^#^
Yang et al. [53]	May 2020	China	Retrospective	ICU mortality at 28 days	Incidence of ARDS and proportion of patients requiring mechanical ventilation	52	20/32	Critical ill patients admitted to Wuhan Jin Yin-tan Hospital from December 24, 2019, to January 26, 2020	Admission after January 12, 2020, oxygen therapy with FiO_2_ < 60%	7^#^
Yu et al. [54]	May 2020	China	Multicenter, prospective, observational study	Evaluation of outcomes and complications and intensity of treatment	NA	226	NA	All patients admitted in ICU from 8 AM of February 26, 2020, to 8 AM of the next day. The ICUs have to have closed adult units, at least 10 beds, full-time physicians and nurses	NA	7^#^
Zeng et al. [55]	April 2020	China	Case report	Description	NA	1	NA	NA	NA	NA
Zhang et al. [56]	June 2020	China	Retrospective	To analyze epidemiological, clinical, laboratory, and radiological features of patients COVID-19 admitted in Zhongan Hospital of Wuhan University from January 2 to February 10, 2020	NA	221	55/166	NA	NA	7^#^
Zhao et al. [57]	April 2020	China	Retrospective	To explore the characteristics of patients with COVID-19 admitted to Jingzhou Central Hospital from January 16 to February 10, 2020	NA	91	30/61	NA	NA	7^#^
Zhou et al. [58]	March 2020	China	Retrospective, multicenter	To explore risk factors of in-hospital mortality	To describe clinical and laboratory courses during hospitalization	191	54/137	Adult patients with laboratory-confirmed COVID-19 from Jinyintan Hospital and Wuhan Pulmonary Hospital, discharged or died before January 31, 2020	Patients still hospitalized or not confirmed by COVID-19 and without available data	7^#^

Quality of assessment following the 3-item, 5-point Oxford Quality Scale^°^ or the Newcastle-Ottawa Scale^#^ COVID-19

*NA* not applicable, *ICU* intensive care unit, *STEMI* ST-elevation myocardial infarction, *WHO* World Health Organization, *RT-PCR* real-time polymerase chain reaction, *PCI* percutaneous coronary intervention, *CMR* cardiovascular magnetic resonance, *SARS-CoV-2* severe acute respiratory syndrome coronavirus 2, *ARDS* acute respiratory distress syndrome, *FiO*_*2*_ oxygen inspired fraction

**Table 2:** Characteristics and outcomes of retrieved trials for the outcome “Acute cardiac injury” and “Acute myocardial infarction”

Acute cardiac injury^**Δ**^												
Authors	Age (years) all patients	Age (years) treatment group	Age (years) control group	Sex M/F^**+**^	Cardiological comorbidities (n° pts)	Other comorbidities (n° pts)	COVID-19 severity	Acute cardiac injury (n° pts or cases-controls)	Troponin T or I value—BNP or NT-proBNP value^**π**^	LVEDV – EF (n° pts or cases-controls)^**π**^	Other no cardiac complications (n° pts)	Outcome (n° pts or cases-controls)
Bangalore et al. [13]	63 (54–73)^§^	60 (56–73)^§^	66 (54–73)^§^	15/3	Hypertension (11) Diabetes (6) Dyslipidemia (7) Smoking (1) Coronary heart disease (3)	Chronic kidney disease (1)	NA	10	TnI 13.5 (4.8–41) ng/ml^§^ TnT 0.02 ng/ml^§^	Not reported (2)	NA	Death (4-9)
Cao et al. [15]	54 (37–67)^§^	66 (54–76)^§^	31 (35–62)^§^	53/49	Hypertension (28) Diabetes (11) Cardiovascular disease (5) Cerebrovascular diseases (6)	Respiratory diseases (10) Malignancy (4) Chronic kidney disease (4) Chronic liver disease (2)	Patients subjected to mechanical ventilation, ECMO, and CRRT	6–9	NA	NA	Shock (10) ARDS (20) Acute infections (17) Acute kidney damage (20) Chronic kidney damage (34) ECMO (3) Mechanical ventilation (7) CRRT (4)	Death (6-11) Discharge (12-73)
Chen et al. [16]	57.6 (44.9–70.3)^§^	56.1 ± 13.5°	61.7 ± 9.6°	36/18	Hypertension (16) Diabetes (25) Coronary heart disease (6)	NA	Severe condition if: Respiratory distress, RR > 30 acts/min or SpO_2_ < 93% rest or P/F < 300 Critical condition if: Mechanical ventilation or shock or failure of other organs that requires intensive monitoring and treatment	0–3	TnI 0.29 ± 0.68 ng/ml° NT-proBNP 1582 ± 2374 pg/ml°	NA	AKI (18)	NA
Chen et al. [17]	62 (44–70)^§^	68 (62–77)^§^	51 (37–66)^§^	171/103	Hypertension (93) Smoke (12) Diabetes (47) Cardiovascular disease (23) Chronic heart failure (1) Cerebrovascular disease (4)	Chronic lung disease (18) Malignancy (7) Chronic kidney disease (4) Autoimmune disease (2)	According to the China Guidance for Corona Virus Disease 2019 (6th edition)	72–18	NA	NA	ARDS (192) Sepsis (179) AKI (29) DIC (21) Shock (46) Acute liver injury (13) Mechanical ventilation (119) CRRT (3) ECMO (1)	Death (113) Recovered (161)
Du et al. [22]	65.8 ± 14.2°	NA	NA	62/23	Hypertension (32) Diabetes (19) Coronary heart disease (10) Cerebrovascular diseases (7)	Chronic liver disease (5) Chronic kidney disease (3) COPD (2) Malignancy (6)	On the recommendations by the National Institute for Viral Disease Control and Prevention, China (fifth edition)	38	NA	NA	Respiratory failure (80) Shock (69) ARDS (63) Acute liver injury (38) Sepsis (28)	Death (51)
Guo et al. [25]	58.5 (14.66)°	53.53 (13.22)°	34 (65.4)°	91/96	Smoking (18) Hypertension (61) Coronary heart disease (21) Cardiomyopathy (8) Diabetes (28)	COPD (4) Malignancy (13) Chronic kidney disease (6)	NA	135–52	NT-proBNP 268.4 (75.3–689.1)^§^	NA	ARDS (46) Acute coagulopathy (42) Liver injury (19) Kidney injury (18) Mechanical ventilation (45)	Death (12-31)
Huang et al. [27]	49 (41–58)^§^	49 (41–61)^§^	49 (41–57.5)^§^	30/11	Smoke (3) Hypertension (6) Diabetes (8) Cardiovascular disease (6)	COPD (1) Malignancy (1) Chronic liver disease (1)	Patients developed ARDS, requiring ICU admission and oxygen therapy	4-1	TnI > 28 pg/ml	NA	Pneumonia (41) ARDS (12) AKI (3) Secondary infection (4) Shock (3) CRRT (3) NIV (10) Invasive mechanical ventilation (2) ECMO (2)	Death (6) Discharge (28)
Ruan et al. [39]	NA	67 (15–81)^§^	50 (44–81)^§^	102/48	Hypertension (52) Cardiovascular disease (13) Diabetes (25) Cerebrovascular disease (12)	COPD (3) Chronic kidney disease (2) Malignancy (3) Chronic liver disease (4)	NA	27	NA	NA	Respiratory failure (71) ARDS (62) AKI (23) Infection (12) Mechanical ventilation (25) ECMO (7) CRRT (5)	Death (68) Discharged (82)
Shao et al. [42]	73.5 (29–66)^§^	NA	NA	13/5	Hypertension (10) Diabetes (4) Cerebrovascular disease (5)	Renal disease (3) Malignancy (2) COPD (2) HIV (1) Autoimmune disease (1)	Based on The National Health Commission of China Guidelines (7th edition)	10	NA	NA	ARDS (17) Invasive mechanical ventilation (11) CRRT (7) AKI (7) Shock (6) ECMO (1) Gastrointestinal bleeding (1) Perforation of the intestine (1) Co-infection (5)	Death (10)
Kunal et al. [32].	51.2 ± 17.7°	60.9 ± 15.1°	47.9 ± 17.4°	70/38	Hypertension (41) Diabetes (35) Cardiovascular disease (14) Dyslipidemia (6) Heart failure (1)	COPD (6)	NA	28–80	TnT 0.66 ± 1.28° mcg/L	NA	Sepsis (25) ARDS (12) AKI (8) Diabetic ketoacidosis (3) Intracranial hemorrhage (1)	Death (16-14)
Shi et al. [43]	64 (21–95)^§^	74 (34–95)^§^	60 (21–90)^§^	205/211	Hypertension (127) Diabetes (60) Coronary heart disease (44) Cerebrovascular disease (22) Chronic heart failure (17)	chronic kidney disease (14) COPD (12) Malignancy (9) Hepatitis B infection (4)	NA	82–334	Hs TnI 0,19 (0.08–1.12) mcg/L^§^ NT-proBNP 1689 (698–3327) pg/ml^§^	NA	Noninvasive ventilation (51) Invasive mechanical ventilation (32) CRRT (2) ARDS (97) AKI (8)	Death (42-15) Discharge (2-38) Recovered (38-281)
Shi et al. [44]	63 (50–72)^§^	73 (66–80)^§^	57 (43–70)^§^	322/349	Hypertension (199) Diabetes (97) Coronary heart disease (60) Chronic heart failure (22) Cerebrovascular disease (22) Atrial fibrillation (7)	Chronic renal disease (28) COPD (23) Cancer (23)	Severe and critical diseases are defined by the presence of any of RR > 30 acts/min; oxygen saturation ≤ 93%; PaO_2_/FiO_2_ ratio < 300 mmHg; respiratory failure requiring mechanical ventilation; shock; respiratory failure with other organ failure requiring ICU management	106	TnI 0.159 (0.075–0.695) ng/ml^§^ NT-proBNP 1346 (474–3018) pg/ml^§^	NA	Invasive mechanical ventilation (36) ECMO (2) CRRT (4)	Death (62) Survivors (609)
Szekely et al. [47]	66.1 ± 17.3°	65.9 ± 20°	69.8 ± 16°	63/37	Hypertension (57) Ischemic heart disease (16) Congestive heart failure (7) Cerebrovascular disease (11) Atrial fibrillation (715)	Chronic renal disease (10) Diabetes (29) Obesity (29) Asthma (7) Malignancy (5)	Acuity score	Troponin level above the 99th percentile was reported in 20% of the patients	Tn I 11 (5–39) ng/L^§^ BNP 43 (18–144) pg/L^§^	EF 58.2 ± 4°	NA	NA
Tavazzi et al. [48]	69^+^	NA	NA	1/0	NA	NA	NA	1	NA	LVEDV 56 mm^+^ EF 34%^+^	Bilateral interstitial pneumonia (1) Mechanical ventilation (1) IABP (1) ECMO (1) Septic shock (1)	Death (1)
Wan et al. [50]	47 (36–55)^§^	44 (33–49)^§^	56 (52–73)^§^	72/63	Current smoking (9) Diabetes (12) Hypertension (13)	Malignancy (4) Pulmonary disease (1) Chronic liver disease (2)	The mild group had mild symptoms and no pneumonia; the normal group had fever, respiratory symptoms, and pneumonia; the severe group had respiratory distress, RR ≥ 3acts/min at rest, mean oxygen saturation ≤ 93%, and P/F ≤ 300 mmHg; the critical group had respiratory failure with mechanical ventilation, shock, and other failures, requiring ICU treatment	8-2	NA	NA	ARDS (21) AKI (5) Secondary infection (7) Shock (1) CRRT (5) Invasive mechanical ventilation (1)	Death (0-1) Discharge (10-5) Hospitalization (85-35)
Xiong et al. [52]	58.5 (47–69)^§^	56 (37–64)^§^	64 (53–76)^§^	80/63	Diabetes (19) Hypertension (45) Coronary Heart Disease (17) Cardiovascular disease (8)	COPD (1) Malignancy (4) Chronic liver disease (2)	Moderate cases: mild respiratory symptoms with pneumonia Severe cases: dyspnea, RR ≥ 30/min, blood oxygen saturation ≤ 93%, PaO_2_/FiO_2_ ratio ≤ 300 mmHg, and/or pulmonary inflammation progressing > 50% within 24 to 48 h. Critical cases: respiratory failure, shock, and/or multiple organ dysfunction	4–19	TnT > 0.02 ng/ml^+^ NT-proBNP 71.5 (27–363.5)^§^ pg/ml	NA	Shock (16) ARDS (20) Liver dysfunction (15) AKI (3) Invasive mechanical ventilation (10) ECMO (3)	Discharge (61-48) Death (0-7)
Yang et al. [53]	59.7 (13,3)°	51,9 (12.9)°	64.6 (11.2)°	14/6	Chronic cardiac disease (5) Cerebrovascular disease (7) Diabetes (9) Smoke (2)	Chronic pulmonary disease (4) Malignancy (2)	Admission to ICU requiring mechanical ventilation or FiO_2_ ≥ 60%	3–9	TnI > 28 pg/ml	NA	ARDS (35) AKI (15) Liver dysfunction (15) Hospital-acquired pneumonia (6) Invasive mechanical ventilation (22) ECMO (6) CRRT (9)	Death (32) Discharge (20)
Yu et al. [54]	64 (57–70)^§^	NA	NA	139/87	Hypertension (96) Coronary heart disease (22) Myocardial infarction (6) Congestive heart failure (4) Diabetes (47) Cerebrovascular disease (15)	Chronic pulmonary disease (15) Chronic hepatopathy (3) Chronic nephrosis (8) Malignancy (10)	RR > 40 acts/min, PaO_2_ < 60 mmHg, SpO_2_ < 90% with oxygen support of ≥7 L/min for at least 30 min, PaCO_2_ > 50 mmHg, hemodynamic instability, use of vasopressors, GCS ≤ 12, CRRT	61	TnI > 0.3 ng/mL	NA	ARDS (161) Shock (36) AKI (57) Bacterial or fungal infection (49) Gastrointestinal hemorrhage (7)	Death (9) Discharge from ICU (13) ICU (204)
Zhang et al. [56]	55 (39–66.5)^§^	62 (52–74)^§^	51 (36–64.3)^§^	108/113	Hypertension (54) Diabetes (22) Cardiovascular disease (22) Cerebrovascular disease (15)	COPD (6) Chronic kidney disease (6) Chronic liver disease (7) Malignancy (9)	Admission in ICU	16-1	NA	NA	ARDS (48) Shock (15) AKI (10) CRRT (5) Mechanical ventilation (16) ECMO (10)	Death (12-0) Discharge (7-35) Hospitalization (36-131)
Zhao et al. [57]	46^§^	50.5^§^	42^§^	49/42	Hypotension (18) Diabetes (3)	COPD (1) Autoimmune disease (1) Kidney disease (1) Malignancy (3)	NA	8–6	NA	NA	Pneumonia (91) Digestive tract disease (14) Liver disease (18) Renal disease (5) Coagulopathy (19) Mechanical ventilation (5) CRRT (3)	Death (2-0) Discharge (14-2) Hospitalization (26-49)
Zhou et al. [58]	56 (46–67)^§^	69 (63–76)^§^	52 (45–58)^§^	119/72	Hypertension (58) Diabetes (36) Coronary heart disease (15)	COPD (6) Carcinoma (2) Chronic kidney disease (2)	NA	32-1	TnI > 28 pg/ml	NA	Sepsis (112) Respiratory failure (103) ARDS (59) Invasive mechanical ventilation (32) Coagulopathy (37) Septic shock (38) Acute kidney injury (28) ECMO (3) CRRT (10) Secondary infection (28)	Death (54) Discharged (137)
**Acute myocardial infarction**												
**Authors**	**Age (years) all patients**	**Age (years) treatment group**	**Age (years) control group**	**Sex M/F**^**+**^	**Cardiological comorbidities (n° pts)**	**Other comorbidities (n° pts)**	**COVID-19 severity**	**EKG (n° pts)**	**Coronarography/PCI (n° pts or cases-controls)**	**Troponin T or I value -BNP or NT-proBNP value**^**π**^	**Other no cardiac complications (n° pts)**	**Outcome (n° pts or cases-controls)**
Bangalore et al. [13]	63 (54–73)^§^	60 (56-73)^§^	66 (54-73)^§^	15/3	Hypertension (11) Diabetes (6) Dyslipidemia (7) Smoking (1) Coronary heart disease (3)	Chronic kidney disease (1)	NA	STEMI (8)	6/5–3/0	TnT 6.3 (5.3–7.2) ng/ml^§^ TnI 91 (65.5–345) ng/ml^§^	NA	Death (4-9)
Barton et al .[14]	77/42^+^	77	42	2/0	Hypertension (2) Obesity (2) Coronary heart disease (2) Dilated cardiopathy (1)	Oncocytoma (1) Prostate hyperplasia (1) Muscular dystrophy (1) Cirrhosis (1) Nephrosclerosis (1)	NA	NA	NA	NA	ARDS (1) Liver failure (1) Pneumonia ab ingestis (1)	Death (2)
Dominguez-Erquicia et al. [20]	64^+^	NA	NA	1/0	NA	NA	NA	STEMI (1)	1/1	NA	Bilateral pneumonia (1)	Discharge (1)
Dweck et al. [24]	62 (52–71)^§^	64 (53–73)^§^	60 (51–69)^§^	844/365	Hypertension (445) Diabetes (233) Ischemic heart disease (167) Heart failure (113) Valvular disease (80)	NA	Patients in critical care setting (ICU, coronary care unit, emergency room, cardiac catheter laboratory)	Not reported (27) STEMI (8)	NA	NA	NA	NA
Kim HN et al. [30]	60^+^	NA	NA	1/0	Hypertension (1) Diabetes (1) Dyslipidemia (1)	NA	Mechanical oxygenation supports	STEMI (1)	1/1	TnI 46.1 ng/ml^+^ NT-proBNP 1971.5 pg/ml^+^	Bilateral infiltration (1) Mechanical ventilation (1) CRRT (1) ECMO (1)	Death (1)
Kunal et al. [32]	51.2 ± 17.7°	60.9 ± 15.1°	47.9 ± 17.4°	70/38	Hypertension (41) Diabetes (35) Cardiovascular disease (14) Dyslipidemia (6) Heart failure (1)	COPD (6)	NA	STEMI (3) NSTEMI (1)	NA	NA	Sepsis (25) ARDS (12) AKI (8) Diabetic ketoacidosis (3) Intracranial hemorrhage (1)	Death (16-14)
Stefanini et al. [46]	68 ± 11°	NA	NA	20/8	Hypertension (20) Diabetes (9) Previous myocardial infarction (3)	Chronic kidney disease (8)	NA	STEMI (25) New LBBB (3)	28/17	NA	NA	Death (11) Discharge (16) ICU (1)

Data are presented as real number^+^, percentage^^^, mean ± SD^°^, or median and (IQR)^§^

*M* male, *F* female, *pts* patients, *TnT* troponin T, *TnI* troponin I, *BNP* brain natriuretic peptide, *NT-proBNP* N-terminal pro-brain natriuretic peptide, *EF* ejection fraction, *LVEDV* left ventricular end-diastolic volume, *NA* not applicable, *ECMO* extra-corporeal membrane oxygenation, *CRRT* continuous renal replacement therapy, *ARDS* acute respiratory distress syndrome, *RR* respiratory rate, *SpO*_*2*_ peripheral oxygen saturation, *P/F* PaO_2_/FiO_2_, *PaO*_*2*_ partial pressure of oxygen in arterial blood, *FiO*_*2*_ oxygen inspired fraction, *PaCO*_*2*_ pressure of arterial carbon dioxide, *AKI* acute kidney injury, *COPD* chronic obstructive pulmonary disease, *ICU* intensive care unit, *NIV* noninvasive ventilation, *HIV* human immunodeficiency virus, *IABP* intra-aortic balloon pump, *PCI* percutaneous coronary intervention, *EKG* electrocardiogram, *STEMI* ST-elevation myocardial infarction, *DIC* disseminated intravascular coagulation, *LBBB* left bundle branch block

^Δ^As reported in studies, not better specified; ^π^referred to patient with complication in exam

**Table 3:** Characteristics and outcomes of retrieved trials for outcome “Takotsubo syndrome”

Takotsubo syndrome												
Authors	Age (years) all patients	Age (years) treatment group	Age (years) control group	Sex (M/F)^**+**^	Cardiological comorbidities (n° pts)	Other comorbidities (n° pts)	COVID-19 severity	Type of CMP (n° pts or cases-controls)	Troponin T or I values – BNP or NT-proBNP values ^**π**^	EF value (n° pts)^**π**^	Other no cardiac complications (n° pts)	Outcome (n° pts or cases-controls)
Dabbagh et al. [18]	63^+^	NA	NA	0/1	Dilated cardiomyopathy (1) Chronic heart failure (1)	NA	NA	Takotsubo syndrome (1)	Tn I 2410 ng/L^+^	40%	NA	Discharge (1)
Dweck et al. [24]	62 (52–71)^§^	64 (53–73)^§^	60 (51–69)^§^	844/365	Hypertension (445) Diabetes (233) Ischemic heart disease (167) Heart failure (113) Valvular disease (80)	NA	Patients in critical care setting (ICU, coronary care unit, emergency room, cardiac catheter laboratory)	Takotsubo syndrome (19)	NA	NA	NA	NA
Meyer et al. [34]	83^+^	NA	NA	0/1	Hypertension (1)	NA	NA	Takotsubo syndrome (1)	TnT 1142 ng/L^+^	NA	Pneumonia (1)	Discharge (1)
Sala et al. [40]	43^+^	NA	NA	0/1	NA	NA	NA	Inverted Takotsubo syndrome (1)	TnT 135 ng/L^+^ NT-proBNP 512 pg/mL^+^	EF 43%	NA	NA
Solano-Lopez et al. [45]	50^+^	NA	NA	1/0	NA	Mediastinal tumor (1)	NA	Inverted Takotsubo syndrome (1)	TnI 64 pg/ml^+^ BNP 790 pg/ml^+^	NA	Pneumonia (1)	Discharge (1)
Stӧbe et al.	64 ± 19.1°	71 ± 15.2°	41 ± 11.8°	14/4	Hypertension (13) Paroxysmal atrial fibrillation (4) Coronary artery disease (2) Dyslipidemia (4) Diabetes (5) Stroke (3)	Chronic kidney disease (7) COPD (1)	Severe symptoms: mechanical ventilation needed	Inverted Takotsubo (7/1)	TnT 36 ± 23 pg/ml° NT-proBNP 1724 ± 1058 pg/ml°	62 ± 6.5%° (18)	Mechanical ventilation (14)	NA

Data are presented as real number^+^, percentage^^^, mean ± SD^°^, or median and (IQR)^§^

*M* male, *F* female, *pts* patients, *CMP* cardiomyopathy, *TnT* troponin T, *TnI* troponin I, *BNP* brain natriuretic peptide, *NT-proBNP* N-terminal pro-brain natriuretic peptide, *EF* ejection fraction, *LVEDV* left ventricular end-diastolic volume, *NA* not applicable, *ICU* intensive care unit, *COPD* chronic obstructive pulmonary disease

^Δ^As reported in studies, not better specified; ^π^referred to patient with complication in exam

**Table 4:** Characteristics and outcomes of retrieved trials for the outcome “Myocarditis”

Myocarditis													
Authors	Age (years) all patients	Age (years) treatment group	Age (years) control group	Sex M/F^**+**^	Cardiological comorbidities (n° pts)	Other comorbidities (n° pts)	COVID-19 severity	N° pts o cases-controls with myocarditis	Troponin T or I value -BNP or NT-proBNP value^**π**^	LVEDV – EF (n° pts or cases-controls)^**π**^	Inotropes and corticosteroids	Other no cardiac complications (n° pts)	Outcome (n° pts or cases-controls)
Deng et al. [19]	65 (49–70.8)^§^	68 (57–77)^§^	56 (39–67)^§^	57/55	Hypertension (36) Diabetes (19) Coronary heart disease (15) Atrial fibrillation (4)	COPD (4)	Severe patients if: Respiratory distress and RR> 30 acts/min or SpO2 < 93% or P/F < 300 Critical patients if: Respiratory failure with mechanical ventilation or shock or multi-organ failure that require hospitalization in UTI	13-1	TnI > 0.12 ng/ml^+^ NT-proBNP 1142 ng/ml (388.3–5956.5)^§^	NA	NA	Mechanical ventilation (28) ECMO (3)	Death (14) Discharge (37)
Dweck et al. [24]	62 (52–71)^§^	64 (53–73)^§^	60 (51–69)^§^	844/365	Hypertension (445) Diabetes (233) Ischemic heart disease (167) Heart failure (113) Valvular disease (80)	NA	Patients in critical care setting (ICU, coronary care unit, emergency room, cardiac catheter laboratory)	35	NA	Low EF (21)	NA	NA	NA
Doyen et al. [21]	69^+^	NA	NA	1/0	Hypertension (1)	NA	NA	1	TnI 9002 ng/L^+^	Normal EF	Hydrocortisone	Mechanical ventilation (1)	Discharge (1)
Hu et al. [26]	37^+^	NA	NA	1/0	NA	NA	NA	1	TnT > 10,000 ng/L^+^ BNP > 21,025 ng/L^+^	LVEDV 58 mm^+^ EF 27% ^+^	Milrinone Methylprednisolone Immunoglobulins	Pneumonia (1) Pleural effusion (1)	Discharge (1)
Hussain et al. [28]	51^+^	NA	NA	1/0	Hypertension (1)	NA	NA	1	Tn 0.29 ng/ml^+^ BNP 1287 pg/ml^+^	EF 20%	Dobutamine	ARDS (1) Mechanical ventilation (1)	Death (1)
Inciardi et al. [29]	52^+^	NA	NA	0/1	NA	NA	NA	1	TnT 0.24 ng/ml^+^ NT-proBNP 5647 pg/ml^+^	EF 35%	Dobutamine Methylprednisolone	Not reported	Hospitalized (1)
Kim IC et al. [31]	21^+^	NA	NA	0/1	NA	NA	NA	1	TnI 1,26 ng/ml^+^ NT-proBNP 1929 pg/mL^+^	Severe dysfunction EF	NA	NA	NA
Kunal et al. [32]	51.2 ± 17.7°	60.9 ± 15.1°	47.9 ± 17.4°	70/38	Hypertension (41) Diabetes (35) Cardiovascular disease (14) Dyslipidemia (6) Heart failure (1)	COPD (6)	NA	3	NA	NA	NA	Sepsis (25) ARDS (12) AKI (8) Diabetic ketoacidosis (3) Intracranial hemorrhage (1)	Death (1) Recovery (2)
Monmeneu et al. [35]	43^+^	NA	NA	1/0	NA	NA	NA	1	TnT 29 ng/L^+^ NT-proBNP 456 pg/ml^+^	EF 50–55%	Methylprednisolone	Pneumonia (1) Pleural effusion (1)	Discharge (1)
Pascariello et al. [36]	19^+^	NA	NA	1/0	NA	Autistic spectrum disorder (1)	NA	1	TnT 1033 ng/L^+^ NT-proBNP 47,650 ng/L^+^	LVEDV 56 mm^+^ EF 15–20%^+^	Adrenaline Dexamethasone	Bilateral interstitial pneumonia (1) Mechanical ventilation (1)	Discharge (1)
Paul et al. [37]	35^+^	NA	NA	1/0	NA	NA	NA	1	TnI 2885 ng/L	Normal EF	NA	NA	Discharge (1)
Zeng et al. [55]	63^+^	NA	NA	1/0	Smoke (1)	Allergic cough (1)	NA	1	TnI 11.37 g/L^+^ NT-proBNP 22,600 pg/mL^+^	LVEDV 61 mm^+^ EF 32%^+^	Methylprednisolone Immunoglobulins	ARDS (1) CRRT (1) ECMO (1) Septic shock (1)	Death (1)

Data are presented as real number^+^, percentage^^^, mean ± SD^°^, or median and (IQR)^§^

*M* male, *F* female, *pts* patients, *TnT* troponin T, *TnI* troponin I, *BNP* brain natriuretic peptide, *NT-proBNP* N-terminal pro-brain natriuretic peptide, *EF* ejection fraction, *LVEDV* left ventricular end-diastolic volume, *COPD* chronic obstructive pulmonary disease, *RR* respiratory rate, *SpO*_*2*_ peripheral oxygen saturation, *P/F* PaO_2_/FiO_2_, *PaO*_*2*_ partial pressure of oxygen in arterial blood, *FiO*_*2*_ oxygen inspired fraction, *NA* not applicable, *ECMO* extra-corporeal membrane oxygenation, *ARDS* acute respiratory distress syndrome, *AKI* acute kidney injury, *CRRT* continuous renal replacement therapy

^Δ^As reported in studies, not better specified; ^π^referred to patient with complication in exam

**Table 5:** Characteristics and outcomes of retrieved trials for the outcome “Pericardial Effusion”

Pericardial effusion											
Authors	Age (years) all patients	Age (years) treatment group	Age (years) control group	Sex M/F^**+**^	Cardiological comorbidities (n° pts)	Other comorbidities (n° pts)	COVID-19 severity	Volume (n° pts or cases-controls)	Cardiac tamponade (n° pts or cases/controls)	Other no cardiac complications (n° pts)	Outcome (n° pts or cases- controls) Outcome (n° pts or cases-controls)
Chen et al. [16]	57.6 (44.9–70.3)^§^	56.1 ± 13.5 °	61.7 ± 9.6°	36/18	Hypertension (16) Diabetes (25) Coronary heart disease (6)	NA	Severe condition: Respiratory distress, RR > 30 acts/min or SpO_2_ < 93% at rest or P/F < 300 Critical condition if: Mechanical ventilation or shock or failure of other organs that requires monitoring and treatment in ICU	Not reported (1–5)	0	AKI (18)	NA
Dabbagh et al. [18]	63^+^	NA	NA	0/1	Dilated cardiomyopathy (1) Chronic heart failure (1)	NA	NA	800 ml^+^ (1)	1	NA	Discharge (1)
Deng et al. [19]	65 (49–70.8)^§^	56 (39–67)^§^	68 (57–77)^§^	57/55	Hypertension (36) Diabetes (19) Coronary heart disease (15) Atrial fibrillation (4)	COPD (4)	Severe patients if: Respiratory distress and RR> 30 acts/min or SpO_2_ < 93% or P/F < 300 Critical patients if: Respiratory failure with ventilation mechanical or shock or multi-organ failure requiring hospitalization in ICU	6.2 ± 1.1 mm° (3–19)	0	Mechanical ventilation (28) ECMO (3)	Death (14) Discharge (37)
Dweck et al. [24]	62 (52–71)^§^	64 (53–73)^§^	60 (51–69)^§^	844/365	Hypertension (445) Diabetes (233) Ischemic heart disease (167) Heart failure (113) Valvular disease (80)	NA	Patients in critical care setting (ICU, coronary care unit, emergency room, cardiac catheter laboratory)	Not reported (11)	11	NA	NA
Kunal et al. [32]	51.2 ± 17.7°	60.9 ± 15.1°	47.9 ± 17.4°	70/38	Hypertension (41) Diabetes (35) Cardiovascular disease (14) Dyslipidemia (6) Heart failure (1)	COPD (6)	NA	Not reported (2)	0	Sepsis (25) ARDS (12) AKI (8) Diabetic ketoacidosis (3) Intracranial hemorrhage (1)	NA

Data are presented as real number^+^, percentage^^^, mean ± SD^°^, or median and (IQR)^§^

*M* male, *F* female, *pts* patients, *RR* respiratory rate, *SpO*_*2*_ peripheral oxygen saturation, *P/F* PaO_2_/FiO_2_, *PaO*_*2*_ partial pressure of oxygen in arterial blood, *FiO*_*2*_ oxygen inspired fraction, *AKI* acute kidney injury, *NA* not applicable, *ECMO* extra-corporeal membrane oxygenation, *ICU* intensive care unit, *ARDS* acute respiratory distress syndrome, *COPD* chronic obstructive pulmonary disease

^Δ^As reported in studies, not better specified; ^π^referred to patient with complication in exam

**Table 6:** Characteristics and outcomes of retrieved trials for the outcome “Arrhythmias”

Arrhythmias										
Authors	Age (years) all patients	Age (years) treatment group	Age (years) control group	Sex M/F^**+**^	Cardiological comorbidities (n° pts)	Other comorbidities (n° pts)	COVID-19 severity	Type of arrhythmia (n° pts or cases-controls)	Other no cardiac complications (n° pts)	Outcome (n° pts or cases-controls)
Amaratunga et al. [11]	55/60/78/73^+^	NA	NA	2/2	Coronary heart disease (2) Hypertension (2) Dyslipidemia (2) Aortic stenosis (1)	Hyperthyroidism (2)	Severe acute respiratory failure hypoxic, resulting intubation, and mechanical ventilation	Sinus Bradycardia (4)	NA	NA
Cao et al. [15]	54 (37–67)^§^	66 (54–76)^§^	31 (35–62)^§^	53/49	Hypertension (28) Diabetes (11) Cardiovascular disease (5) Cerebrovascular diseases (6)	Respiratory diseases (10) Malignancy (4) Chronic kidney disease (4) Chronic liver disease (2)	Patients subjected to mechanical ventilation, ECMO, and CRRT	Unspecified (7–11)	Shock (10) ARDS (20) Acute infections (17) Acute kidney damage (20) Chronic kidney damage (34) ECMO (3) Mechanical ventilation (7) CRRT (4)	Death (6–11) Discharge (12–73)
Chen et al. [16]	57.6 (44.9–70.3)^§^	56.1 ± 13.5 °	61.7 ± 9.6°	36/18	Hypertension (16) Diabetes (25) Coronary heart disease (6)	NA	Severe condition if: Respiratory distress, RR > 30 acts/min or SpO_2_ < 93% rest or P/F < 300 Critical condition if: Mechanical ventilation or shock or failure of other organs that requires intensive monitoring and treatment	Sinus tachycardia (38) Premature beatings (10) Ventricular tachycardia (3) Atrial fibrillation (1) AV block (2) Sinus Bradycardia (3)	AKI (18)	NA
Deng et al. [19]	65 (49–70.8)^§^	56 (39–67)^§^	68 (57–77)^§^	57/55	Hypertension (36) Diabetes (19) Coronary heart disease (15) Atrial fibrillation (4)	COPD (4)	Severe patients if: Respiratory distress and RR > 30 acts/min or SpO_2_ < 93% or P/F < 300 Critical patients if: Respiratory failure with ventilation mechanical or shock or multi-organ failure requiring hospitalization in ICU	Tachycardia (11–22)	Mechanical ventilation (28) ECMO (3)	Death (14) Discharge (37)
Du et al. [22]	65.8 ± 14.2°	NA	NA	62/23	Hypertension (32) Diabetes (19) Coronary heart disease (10) Cerebrovascular diseases (7)	Chronic liver disease (5) Chronic kidney disease (3) COPD (2) Malignancy (6)	On the recommendations by the National Institute for Viral Disease Control and Prevention, China (fifth edition)	Unspecified (51)	Respiratory failure (80) Shock (69) ARDS (63) Acute liver injury (38) Sepsis (28)	Death (51)
Dweck et al. [24]	62 (52–71)^§^	64 (53–73)^§^	60 (51–69)^§^	844/365	Hypertension (445) Diabetes (233) Ischemic heart disease (167) Heart failure (113) Valvular disease (80)	NA	Patients in critical care setting (ICU, coronary care unit, emergency room, cardiac catheter laboratory)	Ventricular arrhythmia (33–5)	NA	NA
Guo et al. [25]	58.5 (14.66)°	53.53 (13.22)°	34 (65.4)°	91/96	Smoking (18) Hypertension (61) Coronary heart disease (21) Cardiomyopathy (8) Diabetes (28)	COPD (4) Malignancy (13) Chronic kidney disease (6)	NA	Ventricular tachycardia/ventricular fibrillation (2–9)	ARDS (46) Acute coagulopathy (42) Liver injury (19) Kidney injury (18) Mechanical ventilation (45)	Death (12-31)
Kunal et al. [32]	51.2 ± 17.7°	60.9 ± 15.1°	47.9 ± 17.4°	70/38	Hypertension (41) Diabetes (35) Cardiovascular disease (14) Dyslipidemia (6) Heart failure (1)	COPD (6)	NA	Ventricular tachycardia/ventricular fibrillation (2) Sinus bradycardia (1)	Sepsis (25) ARDS (12) AKI (8) Diabetic ketoacidosis (3) Intracranial hemorrhage (1)	NA
Peigh et al. [38]	70–81^+^	NA	NA	1/1	Ascending aorta aneurism (1) Hypertension (1)	Obstructive sleep apnea (1)	Mechanical ventilation	Sinus bradycardia (2) First-degree AV block (1)	Respiratory failure and mechanical ventilation (2)	NA
Seecheran et al. [41]	46^+^	NA	NA	1/0	NA	NA	NA	Atrial flutter (1) Atrial fibrillation (1)	NA	Discharge (1)
Villanueva et al. [49]	68^+^	NA	NA	1/0	Atrial flutter (1) Obesity (1) Hypertension (1) Non-ischemic cardiomyopathy (1) Pacemaker (1) Heart failure (1) Diabetes (1)	Chronic kidney disease stage 3 (1)	NA	Atrial fibrillation (1)	Interstitial pneumonia (1) Mechanical ventilation (1)	NA
Yu et al. [54]	64 (57-70)^§^	NA	NA	139/87	Hypertension (96) Coronary heart disease (22) Myocardial infarction (6) Congestive heart failure (4) Diabetes (47) Cerebrovascular disease (15)	Chronic pulmonary disease (15) Chronic hepatopathy (3) Chronic nephrosis (8) Malignancy (10)	RR > 40 acts/min, PaO_2_ < 60 mmHg, SpO_2_ < 90% with oxygen support of ≥7 L/min for at least 30 min, PaCO_2_ > 50 mmHg, hemodynamic instability, use of vasopressors, GCS ≤ 12, CRRT	Atrial fibrillation (18) Supraventricular tachycardia (2) Ventricular tachycardia (1)	ARDS (161) Shock (36) AKI (57) Bacterial or fungal infection (49) Gastrointestinal hemorrhage (7)	Death (9) Discharge from ICU (13) ICU (204)
Zhang et al. [56]	55 (39–66.5)^§^	62 (52-74)^§^	51 (36–64.3)^§^	108/113	Hypertension (54) Diabetes (22) Cardiovascular disease (22) Cerebrovascular disease (15)	COPD (6) Chronic kidney disease (6) Chronic liver disease (7) Malignancy (9)	Admission in ICU	Unspecified (22-2)	ARDS (48) Shock (15) AKI (10) CRRT (5) Mechanical ventilation (16) ECMO (10)	Death (12-0) Discharge (7-35) Hospitalization (36-131)

Data are presented as real number^+^, percentage^^^, mean ± SD^°^, or median and (IQR)^§^

*M* male, *F* female, *pts* patients, *NA* not applicable, *ECMO* extra-corporeal membrane oxygenation, *CRRT* continuous renal replacement therapy; *ARDS* acute respiratory distress syndrome, *RR* respiratory rate, *SpO*_*2*_ peripheral oxygen saturation, *P/F* PaO_2_/FiO_2_, *PaO*_*2*_ partial pressure of oxygen in arterial blood, *FiO*_*2*_ oxygen inspired fraction, *AV* atrioventricular, *AKI* acute kidney injury, *COPD* chronic obstructive pulmonary disease, *PaCO*_*2*_ pressure of arterial carbon dioxide, *GCS* Glasgow coma scale, *ICU* intensive care unit

^Δ^As reported in studies, not better specified; ^π^referred to patient with complication in exam

For all patients who were reported to have cardiovascular complications, we analyzed demographics (age, sex), risk factors/comorbidities, clinical features (cardiac biomarkers, ECG, and/or echocardiographic findings), and their relation with outcome. A major limitation of this review includes the heterogeneity in cardiac injury definition (ECG/biomarkers). The authors of this systematic review reported the definition provided by the authors for each article included.

### Statistical analysis

The Cochrane handbook for systematic reviews of interventions [59] and Hozo and coll [60]. recommendations were followed in order to perform the meta-analysis. Secondary outcome data were extracted only from the published articles retrieved. Publication bias was evaluated by analyzing the funnel plots. We calculated risk ratio (RR) and 95% confidence interval (CI) to summarize continuous data. A random-effects model was applied to analyze the data. The heterogeneity of the retrieved trials was evaluated through the *I*^2^ statistic. *I*^2^ values above 75% reflected a high heterogeneity [61, 62]. Subgroup analyses were performed as sensitivity analysis according to the type of cardiac complications (acute cardiac injury, cardiogenic shock, heart failure, arrhythmia, right ventricular dysfunction). All statistical analyses were performed using Review Manager (RevMan; Computer program. Version 5.3 Copenhagen: The Nordic Cochrane Centre, The Cochrane Collaboration, 2012).

## Results

**Fig. 2:**
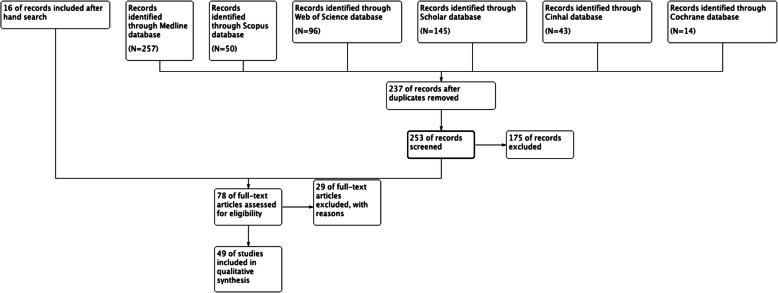
PRISMA flowchart summarizing the literature search strategy

Electronic literature searches identified 605 articles, and 16 further citations were found by hand searching the reference lists of the studies. We initially excluded articles as not relevant for the aim of the review basing on the abstract. We selected 78 articles for full-text review. Twenty-nine studies were further excluded for one of the following reasons: outcome was not reported, preliminary studies, and technical descriptions. Our review finally included 49 clinical studies (Fig. 2). Study characteristics and outcomes are listed in Table 1.

The current study was performed between January and September.

Twenty-two articles (%) were conducted by Chinese groups [15–17, 19, 22, 23, 25–27, 39, 42–44, 50–58], nine (%) were carried out in the USA [11–14, 18, 28, 33, 38, 49], thirteen (%) were from European groups [20, 21, 24, 29, 34–37, 40, 45, 46, 48, 63], two (%) from Korea [30, 31], and one (%) respectively from Trinidad and Tobago [41], Israel [47], and India [32]. Twenty-three articles (%) were case reports or case series [11–14, 18, 20, 21, 26, 28–31, 34–38, 40, 41, 45, 48, 49, 55], twenty (%) were retrospective in nature [15–17, 19, 22, 25, 27, 33, 39, 42–44, 46, 50–53, 56–58], five (%) prospective studies [23, 32, 47, 54, 63], and one international survey [24]. Quality assessments for the included trials are reported in Table 1. The first aim of twenty-seven studies was the description of cardiovascular manifestation in patients with COVID-19 [11, 13, 16, 19, 21, 25, 26, 28, 30–35, 37, 38, 40, 41, 43–46, 48, 49, 51, 55]. The majority of these trials highlighted the impact of COVD-19 on the cardiovascular system and the need for continuous monitoring and prompt treatment. The evaluation of echocardiography was the first aim of one international survey [24] and two prospective studies [47, 63]. Echocardiographic abnormalities were observed in more than half of COVID-19 patients included. The description of the clinical characteristics and/or outcome of patients with COVID-19 was the main aim of 15 articles [12, 15, 17, 22, 23, 27, 39, 42, 50, 52–54, 56–58].

The severity of respiratory involvement was defined as:

- Need for mechanical ventilation or severe acute hypoxic respiratory failure [11, 63]
- Respiratory rate (> 30 breaths/minute), oxygen saturation < 93%, PaO_2_/FiO_2_ < 300, or respiratory failure/shock/multi-organ failure [16, 44, 50, 52]
- Respiratory distress for a respiratory rate > 40 breath/minute, peripheral saturation < 90% and PaO_2_ < 60 mmHg/with 7 l/min, PaCO2 > 50 mmHg, hemodynamic instability or vasopressor therapy, GCS< 12, the need of CRRT [54]
- Clear reference to national and international guidelines [17, 19, 27, 42, 51, 53, 56]

### Primary outcome: cardiac complications and risk factors

#### Acute cardiac injury, acute myocardial infarction, cardiogenic shock, heart failure

Acute cardiac injury was evaluated in 20 articles (as shown in Table 2): 17 retrospectives [15–17, 22, 25, 27, 32, 39, 42–44, 50, 52, 53, 56, 57, 64], two prospective observational studies [47, 54], and one international survey [24]. Heart failure and cardiogenic shock were reported in 10 articles [12, 16, 17, 22, 32, 44, 47, 48, 52, 54]. Acute cardiac injury (ACI) was defined as an increased in cardiac biomarker level [16, 25, 32, 43, 44, 47, 52–54, 57] and/or new abnormalities in ECG/echocardiographic evaluations [17, 22, 27, 58]. Six of the aforementioned studies analyzed cardiac complications in patients with COVID-19 as first objective [16, 32, 43, 44, 48, 52]. Myocardial infarction was evaluated in seven of the papers retrieved (as shown in Table 2): one international survey [24], two retrospective [32, 46], one autopsy report [14], and three case reports [13, 20, 30]. Six out of seven of the aforementioned studies analyzed cardiac complications in patients with COVID-19 as first objective [13, 20, 24, 30, 32, 46].

Characteristics and outcomes of retrieved articles for acute cardiac injury and acute myocardial infarction are listed in Table 2.

In the retrospective study of Kunal et al., the authors enrolled 108 patients with COVID-19 [32] and ACI was defined as serum levels of troponin T above the 99th percentile upper reference limit. The authors found that ACI was the most common CV complication (25.9% of the patients). Heart failure, cardiogenic shock, and acute coronary syndrome were observed in 3.7% of the patients. Patients with ACI were significantly older and had a greater frequency of hypertension, diabetes, and cardiovascular disease in their medical history (*P* = 0.001). The authors also found a statistically significant difference between non-survivor and survivor in patients with acute cardiac injury (*P* = 0.01). No correlation was observed regarding the use of angiotensin-converting enzyme inhibitors or angiotensin receptor blockers and acute cardiac injury. Univariate logistic regression analysis found that acute cardiac injury was a predictor of mortality (OR: 6.28; 95% CI: 2.44–16.17; *P* < 0.0001). Multivariate logistic regression analysis found that acute cardiac injury was the independent predictor of mortality (OR: 11.3; 95% CI: 2.31–55.54; *P* = 0.003). Likewise, in the retrospective study of Guo et al., the authors found cardiac injury in 27.8% of the COVID-19 patients [25]. Cardiac injury was defined as serum levels of troponin T above the 99th percentile upper reference limit. Patients with cardiac injury had a significantly higher rate of hypertension (*P* < 0.001), coronary artery disease (*P* < 0.001), cardiomyopathy (*P* < 0.001), diabetes (*P* < 0.001), chronic obstructive pulmonary disease (*P* = 0.001), and history of ACEI/ARB use (*P* = 0.002). Patients with cardiac injury were more likely to develop complications during hospital stay (i.e., malignant arrhythmia, acute respiratory distress syndrome, acute coagulopathy acute kidney injury). The mortality rate was 37.50% for patients with cardiac injury and 69.44% for patients with cardiac injury and cardiovascular disease in their medical history. Shi et al., in a retrospective study of 416 patients, evaluated the incidence of cardiac complications and the association between cardiac injury and mortality [43]. Cardiac injury was defined as an increase of troponin above the 99th percentile upper reference limit. A diagnosis of acute cardiac injury was made in 82 patients (19.7%). Patients with ACI were older and with comorbidities (i.e., hypertension, coronary heart disease, cerebrovascular disease, chronic heart failure) in comparison to COVID patients without cardiac injury (*P* < 0.001). Troponin and pro-BNP levels were statistically significantly higher in patients with cardiac injury (*P* < 0.001). Even more, in the group of cardiac injury patients, the authors found a higher incidence of invasive mechanical ventilation, use of antibiotics, glucocorticoids, immunoglobulin, acute kidney injury, electrolytes alterations, coagulation disorders, and ARDS. The mortality rate was also higher in this group of patients (*P* < 0.001). Similar findings can be observed in the single-center retrospective observational study by Xiong et al., evaluating the cardiovascular implication in COVID-19 patients [52]. Cardiac injury was defined as an increase in serum troponin T above the upper limit of reference (> 0.02 ng/mL), whereas acute heart failure was defined as the presence of typical symptoms in addition to signs of structural and/or functional cardiac abnormalities. They found that 19.8% had acute cardiac injury and 18.1% had acute heart failure. Acute cardiac injury was the most common complication among COVID-19 patients. The prevalence of cardiac complications was higher in severe cases (*P* = 0.001): severe cases were defined as critical cases presenting respiratory failure, shock, and/or multiple organ dysfunctions. Even more, severe cases were more likely to have comorbidities. Chen et al., in a retrospective study of 113 patients, aimed to relate demographic, clinical, and radiological characteristics between patients recovered versus deceased patients [17]. They found that cardiac complications were more frequent in non-survivor patients in comparison to recovered patients (association between complications and outcomes). In particular, the authors found that 77% of patients with a diagnosis of acute cardiac injury (defined on the basis of cardiac biomarkers and/or ECG alterations) died; half of these patients presented hypertension or cardiological disease in their medical history.

Heart failure (defined on the basis of age-related pro-BNP) was observed in 49% of dead patients: half of these patients presented hypertension or cardiological disease in their medical history. Shi et al., in a retrospective study of 671 patients, evaluated the clinical characteristics and the impact on outcome of cardiac complications in COVID-19 patients [44]. Cardiac injury was defined as an increase of cardiac biomarkers above the 99th percentile upper reference limit. Patients were divided into survivors and non-survivors. The cause of death was attributed to ACI in 30.6% of the patients. Patients with cardiac injury were older and with more comorbidities in comparison to COVID patients without cardiac injury (*P* < 0.001). Acute heart failure represented the cause of death in 19.4% of the patients. Du et al. retrospectively analyzed the risk factors and clinical features of 85 COVID-19 patients who died [22]. ACI was observed in 44.7% of the patients. Cardiac arrest represented 8.64% of cases of death. Acute coronary syndrome represented 4.94% of cases of death. Similar results were reported in the remaining included studies, with the percentage of acute cardiac injury varied from 12 to 55% [15, 27, 42, 53, 54, 56–58]. Contrarily, Wan et al. found that acute cardiac injury was observed in 7.4% of the cases; no statistical difference was found between the percentage of acute cardiac injury in mild and severe forms [50]. The authors examined the clinical feature of COVID-19 patients and divided the enrolled patients into mild and severe forms (respiratory rate > 30 beats/minute, mean oxygen saturation < 93%, PaO_2_/FiO_2_ < 300).

The prospective international study by Dweck et al. reported data from 1216 echocardiographic examinations in COVID-19 patients [24]. The indications for echocardiographic evaluation were suspected left heart failure in 40%, elevated cardiac biomarkers in 26%, right heart failure in 20%, and ST-segment elevation in 9% of the cases. Chest pain and ST-segment elevation represented the indication for performing echocardiographic evaluation in 13% of the cases (14 patients). Severe impairment of the left ventricle was observed in 9% of the cases (112 patients) and of the right ventricle in 6%. New myocardial infarction was observed in 3% of the population examined [24]. Seven percent of these patients exhibited elevated levels of troponin and/or BNP. Similarly, the prospective study of Szekely et al. aimed to perform a comprehensive echocardiographic evaluation of COVID-19 patients [47]. One hundred patients were enrolled of whom 68% showed echocardiographic abnormalities: RV dilatation (39%), LV diastolic dysfunction (16%), and LV systolic dysfunction (20%). Troponin level above the 99th percentile was reported in 20% of the patients. Comorbidities were observed in 72% of the patients.

The retrospective study by Chen et al., on 54 patients, aimed to evaluate cardiac complications on COVID-19 patients and to identify possible risk factors [16]. Patients were divided into critical and severe forms of COVID-19 based on clinical evaluation (i.e., respiratory rate, peripheral oxygen saturation and gas exchange, respiratory failure, mechanical ventilation requirement, organ dysfunction, shock). An echocardiographic scan was performed in 31 patients: new-onset heart failure was observed in 20% of critically ill patients; 13.3% presented with right heart failure and pulmonary hypertension and 6.7% with left heart failure. Severe cardiac injury was found to be an independent risk factor predicting the critical status of COVID-19 patients (OR = 2.4, 95% CI 1.8–20.1, *P* = 0.4).

Concerning acute myocardial infarction, in the retrospective study of Stefanini et al., the authors evaluated the incidence, clinical presentation, angiographic findings, and outcome of 28 COVID-19 patients who underwent urgent coronary angiography for STEMI [46]. STEMI was defined according to ESC guidelines [65]. 71.4% of the population had hypertension in their medical history, 32.1% diabetes mellitus, 28.6% chronic kidney disease, and 10.7% previous myocardial infarction. In 85.7% of the cases, STEMI represented the cause of hospitalization. At echocardiographic evaluation, left ventricular ejection fraction < 50% was observed in 60.7% of the cases, 82.1% presented regional wall abnormalities, 10.7% diffuse hypokinesia, and 7.1% no abnormalities. On coronary angiography evaluation, 60.7% presented a culprit lesion needing revascularization, whereas 39.3% did not present coronary artery disease. No one underwent fibrinolysis. A follow-up evaluation reported that 39.3% of patients died, 3.6% were still in hospital, and 57.1% had been discharged. On the same aspect, Bangalore et al. detected 18 COVID-19 patients with ST-segment: in 8 patients, the diagnosis of myocardial infarction was confirmed (i.e., coronary angiography, echocardiography) [13]. The group of patients with myocardial infarction presented a high incidence of hypertension (86%), hypercholesterolemia (29%), and diabetes mellitus (43%) as risk factors. Even more, the echocardiographic evaluation showed a low ejection fraction in 88% of the cases and regional wall motion abnormality in 75% and a higher level of troponin and D-dimer. Six out of eight patients underwent coronary angiography: 5 patients were treated with PCI. In-hospital mortality occurred in 50% of patients with myocardial infarction.

To summarize, patients with acute cardiac injury seemed to be significantly older, with comorbidities, more likely to develop complications, and with higher mortality rates. Acute cardiac injury represented the prevalent cardiac complications observed in COVID-19 patients (from 20 to 45% of the patients). Acute cardiac injury was found to be an independent risk factor for severe forms of SARS-CoV-2 infection and an independent predictor of mortality.

#### Takotsubo syndrome

The evidence of cardiomyopathies was evaluated in six of the papers retrieved (as shown in Table 3): one international survey [24], one observational study [63], and four case reports [18, 34, 40, 45]. The evaluation of cardiological complications in patients with COVID-19 was the main aim of 5 studies [18, 24, 34, 40, 45].

In the international survey by Dweck et al., the author observed echocardiographic evidence of Takotsubo syndrome (TTS) in 2% of cases (19 patients), associated with an increase in troponins (10 patients) and BNP levels (5 patients) [24]. Similarly, Stöbe et al. performed an echocardiographic evaluation study on COVID-19 patients in order to characterize the cardiac abnormalities in this group of patients [63]. Interestingly, the authors performed myocardial deformation analysis, with the evaluation of global and regional circumferential and radial layer strain deformation. They found that the cardiac abnormalities were high in the COVID-19 patients included. The most common findings were a reduced longitudinal strain (71%), absence or dispersion of basal rotation (43%), and reduced circumferential strain in the mid and basal segments (50%). The authors concluded that in the majority of the patients included the LV dysfunction observed was similar to “reverse basal Takotsubo like syndrome.”

Takotsubo was also described in three case reports [34, 40, 45]. In both cases, chest discomfort, elevation of troponin, and EKG alterations were observed with negative coronary angiography. Similarly, the woman was treated with medical therapy and was discharged at home after the resolution of echocardiographic abnormalities. In the clinical cases described by the Spanish and the Italian group, the authors observed an inverted TTS: akinesia of the basal segments of the left ventricle on echocardiographic evaluation [40, 45].

#### Myocarditis

Myocarditis complications were reported in twelve of the papers retrieved (as shown in Table 4): one international survey [24], two retrospective studies [19, 32], and nine case reports [21, 26, 28, 29, 31, 35–37, 55]. All the aforementioned studies aimed at analyzing cardiac complications in patients with COVID-19.

In the international survey of Dweck et al., the authors observed 35 cases of myocarditis (3% of the population studied) with an associated increase in troponin (19 patients) and BNP levels (13 patients) [24]. In the retrospective study by Deng et al., 14 cases (12.5%) of suspected myocarditis were observed [19]. The authors followed the statement from the American Heart Association for the diagnosis of myocarditis [66]. The patients with suspected myocarditis presented the following clinical characteristic:

-A mean age of 74 years

-Predominantly men (71.4%)

-Comorbidities: hypertension (42.6%), diabetes (28.6%), and coronary artery disease (21.4%)

Fourteen patients presented an increase in cardiac biomarkers (i.e., CK-MB and pro-BNP). Even more, 10 patients presented alteration in echocardiographic evaluation, 2 in EKG, and 2 in both exams. In comparison with 98 patients without suspected myocarditis, the authors observed a statistical difference for age, blood saturation of oxygen shortness of breath, and respiratory rate at admission.

In the retrospective study of Kunal et al., the authors enrolled 108 patients with COVID-19 [32]. Myocarditis was classified based on the level of diagnostic certainty:

-Histological/immunohistological evidence

-Symptoms/ECG findings/cardiac biomarkers/TTE/cardiac MRI

However, no proven data on intra-myocytes active viral replication were found.

#### Pericardial effusion

Pericardial effusion was evaluated in five of the papers retrieved (as shown in Table 5): one international survey [24], three retrospective studies [16, 19, 32], and one case report [18]. All the aforementioned studies aimed at analyzing cardiac complications in patients with COVID-19 patients.

In the prospective international study by Dweck et al., cardiac tamponade represented the indication for performing echocardiographic evaluation in 2% of the cases (20 patients) [24]. Cardiac tamponade was confirmed in 1% of the cases.

In the retrospective study by Chen et al., echocardiographic evaluation was performed in 31 patients; pericardial effusion was observed in 5 critical patients and 1 severe case (*P* < 0.01). Pericardial effusion was found to be an independent risk factor predicting the critical status of COVID-19 patients (OR = 3.5, 95% CI 1.8–15.1, *P* = 0.5) [16]. Kunal et al. reported two cases of pericardial effusion (1.9%): one patient with moderate pleural effusion without cardiac tamponade [32]. In the study by Deng et al., pericardial effusion > 5 mm was observed in 19.6% (22 patients) with a statistically significant difference in the percentage of PE in patients with severe versus non-severe form of COVID pneumonia (28.3 vs. 6.7%, *P* < 0.01) [19]. In 13.4% of patients, the authors also observed signs of pulmonary hypertension (according to the definition of the American Society of Echocardiography and the European Society of Cardiology) [67, 68].

#### Arrhythmias

Arrhythmic complications were evaluated in thirteen of the papers retrieved (as shown in Table 6): one international survey [24], seven retrospective [15, 16, 19, 22, 25, 32, 56], one observational [54], two case reports [41, 49], and two case series [11, 38]. Nine out of eleven studies analyzed cardiac complications in patients with COVID-19 as first objective [11, 16, 19, 24, 25, 32, 38, 41, 49]; for four of these articles [11, 38, 41, 49], the development of arrhythmias was the primary outcome of the study.

In the prospective international study by Dweck et al., ventricular arrhythmia represented the indication for performing echocardiographic evaluation in 3% of the cases (38 patients); 33 of 38 patients presented echocardiographic alterations [24]. Contrarily, Yu et al. observed 9.3% of arrhythmic complications (21patients) mostly represented by atrial fibrillation (%) [54]. Supraventricular tachycardia was observed in 2 patients whereas ventricular tachycardia was found in one patient. In the retrospective study by Chen et al., the authors described several ECG alterations and compared the percentage between severe and critical patients [16]:

-Sinus tachycardia 59% (23 patients) of severe patients versus 100% of the critical group (*P* < 0.1)

- Ventricular tachycardia 13.3% (2 patients) of severe patients versus 2.6% (1) of the critical group (no statistical difference)

- Premature beat 20.5% (8 patients) of severe patients versus 13.3% (2 patients) of the critical group (no statistical difference)

- Atrioventricular block 0% of severe patients versus 13.3% (2 patients) of the critical group (*P* = 0.2)

- Sinus bradycardia 5.1% (2 patients) of severe patients versus 6.7% (1 patient) of the critical group (no statistical difference)

- Atrial fibrillation 0% of severe patients versus 6.7% (1 patient) of the critical group (no statistical difference)

Remarkably, the authors stressed that they did not observe an association between tachycardia and body temperature or oxygen saturation.

Tachycardia was observed in 29.5 % of the cases (33 patients) in the retrospective study by Deng et al. [19]: no statistical difference was observed between severe versus non-severe forms of COVID pneumonia (*P* = 0.34). Zhang et al. reported 22 cases of arrhythmia complications in the severe form of COVID-19 patients (40% vs. 1.2%, *P* < 0.001) [56]. Du et al. reported 60% of arrhythmic complications; in 2.47% of cases, malignant arrhythmia represented the cause of death [22]. Cao et al. found arrhythmic complications in 38.9% of intensive care patients in comparison to 13.1% of non-intensive patients [15]. Guo et al. reported an incidence rate of 7% for VT and VF [25].

In the retrospective case series of Amaratunga et al., the authors highlighted bradycardia as a possible important complication of COVID viral infection [11]. Even more, they suggested that the onset of bradycardia in COVID patients has to be regarded as a possible manifestation of a serious cytokine storm. These patients required closer monitoring for possible cardiological sequelae. The authors also speculated on possible etiologists: hypoxia, inflammatory cytokine storm, and drug integrations. Remarkably, the four cases described presented sinus bradycardia, not associated with high changes in temperature: three out of the four patients were sedated with propofol or dexmedetomidine, but the bradycardia persisted even when the same drugs were discontinued. An important aspect to analyze was the association between bradycardia and hypotension; three patients required the use of vasopressors in order to maintain PAM > 65 mmHg.

#### Right ventricular dysfunction

Right ventricle dysfunction was evaluated in six articles retrieved (Table 7): two retrospective studies [16, 19], one prospective study [47], one international survey [24], 1 observational study [63], and one case report [55].

**Table 7:** Characteristics and outcomes of retrieved trials for the outcome “Right ventricular dysfunction”

***Right ventricular dysfunction***												
Authors	Age (years) all patients	Age (years) treatment group	Age (years) control group	Sex (M/F)^**+**^	Cardiological comorbidities (n° pts)	Other comorbidities (n° pts)	COVID-19 severity	Morphological RV alterations (treatment-control or n° pts)	Systolic dysfunction (treatment-control or n° pts)	sPAP (treatment-control or n° pts)	Other no cardiac complications (n° pts)	Outcome (n° pts or cases - controls)
Chen et al. [60]	57.6 (44.9–70.3)^§^	56.1 ± 13.5 °	61.7 ± 9.6°	36/18	Hypertension (16) Diabetes (25) Coronary heart disease (6)	NA	Severe condition if: Respiratory distress, RR > 30 acts/min or SpO_2_ < 93% rest or P/F < 300 Critical condition if: Mechanical ventilation or shock or failure of other organs that requires intensive monitoring and treatment	Enlargement (0–2)	Failure (0–2)	Elevated (0–2)	AKI (18)	NA
Deng et al. [62]	65 (49–70.8)^§^	68 (57–77)^§^	56 (39–67)^§^	57/55	Hypertension (36) Diabetes (19) Coronary heart disease (15) Atrial fibrillation (4)	COPD (4)	Severe patients if: Respiratory distress and RR> 30 acts/min or SpO2 < 93% or P/F < 300 Critical patients if: Respiratory failure with mechanical ventilation or shock or multi-organ failure that requires hospitalization in UTI	None	TAPSE < 16 mm (0-4)	NA	Mechanical ventilation (28) ECMO (3)	Death (14) Discharge (37)
Dweck et al. [38]	62 (52–71)^§^	64 (53–73)^§^	60 (51–69)^§^	844/365	Hypertension (445) Diabetes (233) Ischemic heart disease (167) Heart failure (113) Valvular disease (80)	NA	Patients in critical care setting (ICU, coronary care unit, emergency room, cardiac catheter laboratory)	Dilated (181)	Mild to severe (313)	Elevated (99)	NA	NA
Stӧbe et al. [36]	64 ± 19.1°	71 ± 15.2°	41 ± 11.8°	14/4	Hypertension (13) Paroxysmal atrial fibrillation (4) Coronary artery disease (2) Dyslipidemia (4) Diabetes (5) Stroke (3)	Chronic kidney disease (7) COPD (1)	Severe symptoms: mechanical ventilation needed	NA	RV GLS mildly reduced (4-2)	None	Mechanical ventilation (14)	NA
Szekely Y et al. [47]	66.1 ± 17.3°	65.9 ± 20°	69.8 ± 16°	63/37	Hypertension (57) Ischemic heart disease (16) Congestive heart failure (7) Cerebrovascular disease (11) Atrial fibrillation (71)	Chronic renal disease (10) Diabetes (29) Obesity (29) Asthma (7) Malignancy (5)	Acuity score	Dilated (39)	RVFAC reduced 38.8 ± 11 (10)	NA	NA	NA
Zeng et al. [44]	63^+^	NA	NA	1/0	Smoke (1)	Allergic cough (1)	NA	NA	Reduced TAPSE (1)	Elevated (1)	ARDS (1) CRRT (1) ECMO (1) Septic shock (1)	Death (1)

Data are presented as real number^+^, percentage^^^, mean ± SD^°^, or median and (IQR)^§^

*M* male, *F* female, *pts* patients, *TnT* troponin T, *TnI* troponin I, *BNP* brain natriuretic peptide, *NT-proBNP* N-terminal pro-brain natriuretic peptide, *EF* ejection fraction, *LVEDV* left ventricular end-diastolic volume, *NA* not applicable, *ECMO* extra-corporeal membrane oxygenation, *CRRT* continuous renal replacement therapy, *ARDS* acute respiratory distress syndrome, *RR* respiratory rate, *SpO*_*2*_ peripheral oxygen saturation, *P/F* PaO_2_/FiO_2_, *PaO*_*2*_ partial pressure of oxygen in arterial blood, *FiO*_*2*_ oxygen inspired fraction, *PaCO*_*2*_ pressure of arterial carbon dioxide, *AKI* acute kidney injury, *COPD* chronic obstructive pulmonary disease, *ICU* intensive care unit, *NIV* noninvasive ventilation, *HIV* human immunodeficiency virus, *IABP* intra-aortic balloon pump, *PCI* percutaneous coronary intervention, *EKG* electrocardiogram, *STEMI* ST-elevation myocardial infarction, *DIC* disseminated intravascular coagulation, *LBBB* left bundle branch block

^Δ^As reported in studies, not better specified; ^π^referred to patient with complication in exam

In the prospective international study by Dweck et al., right ventricle dysfunction was found during echocardiography evaluation in 313 patients (21%), with a mild to moderate impairment in 236 patients (19%) and a severe impairment in 77 patients (6%) [24]. From a morphological perspective, RV was dilated in 181 patients (15%), with D-shape left ventricle in 46 patients (4%). Elevated pulmonary artery pressure (PAPs) was found in 99 patients (8%). The independent predictors of RV failure in patients without pre-existing heart disease were suspected right heart failure (OR 2.65, 95% CI 1.88–3.75) and moderate (OR 2.34, 95% CI 1.32–4.29) or severe COVID-19 symptoms (OR 3.19, 95% CI 1.73–6.10).

In the retrospective study by Chen et al., echocardiographic evaluation was performed in 31 patients. Right ventricular dysfunction, with right heart enlargement/pulmonary hypertension, was observed in 2 critical patients, care; both patients died [16].

Comparing patients with severe and mild symptoms, Stӧbe et al. did not find a statistically significant difference in RV function [63]: global longitudinal strain (GLS) of the free wall was −26.6 ± 5.9% in severe cases vs. −27.5 ± 6.1 % in mild cases (*P* = 0.76). In four out of ten patients with severe symptoms, RV GLS was mildly reduced (between −17 and −23%), with a higher value of troponin T and NT-pro-BNP. Similarly, in two out of four patients with mild symptoms, RV GLS was mildly reduced (between −22 and −23%).

Szekely et al. evaluated right ventricular function analyzing RV fractional area change, tricuspid annular plane systolic excursion, systolic lateral annular velocity, and pulmonic flow acceleration time (AT) velocity [47]. RV dilation was found in 39% of the patients and represented the most common echocardiographic pattern in COVID-19 patients. Increased RV end-diastolic area was significantly associated with mortality (1.14 [HR, 1.01–1.32]; *P* = 0.05 for 1 cm^2^).

#### Risk factors

The following risk factors were highlighted during the literature search:

- Hypertension: the majority of the article reported hypertension as one of the main risk factors in patients with COVID-19. Hypertension was observed in 55.6% of the COVID-19 included in the study of Shao et al. [42]. Cao et al. observed that ICU patients were more probable to suffer from comorbidities and found hypertension in 55% of the cases [15]. Chen et al. found that hypertension was more frequent in deceased patients in comparison to recovered (48% vs.24%) [17]. In the study of Zhang et al., hypertension was observed in 24.4% of the patients included, with a significant difference between severe and non-severe forms of COVID-19 (47.3% versus 16.9%, *P* < 0.001) [56]. In the study of Zhou et al., hypertension was observed in 30% of the patients included, with a significant difference between non-survivor and survivor (48% versus 23%, *P* = 0.0008) [58]. Hypertension was significantly different in patients with cardiac injury in comparison with patients with no cardiac injury in the study of Shi et al. (59.8% versus 23.4%, *P* < 0.001) [43]. Kunal et al. found that hypertension was the most common comorbidity (38%) [32], with a significant difference between non-survivor and survivor (*P* = 0.01) and between patients that developed or not acute cardiac injury during in-hospital stay (*P* < 0.0001). Univariate logistic regression analysis found that hypertension was a predictor of mortality (OR: 2.94; 95% CI: 1.23–7.00; *P* = 0.015). Conversely, Du et al. found that hypertension was high (59.6%); however, the authors missed finding any statistical difference between ICU and non-ICU groups (*P* = 0.580) [23]. Similarly, Huang et al. did not find any statistical difference between ICU and non-ICU groups (*P* = 0.93, hypertension in 15% of the cases) [27]. Wan et al. found hypertension in 9.6% of patients: the percentage of hypertension was similar between severe and mild forms of COVID-19 (10 vs. 9.4%) [50]. Finally, Xiong et al. found that hypertension was high in patients included (38.8%); however, the authors missed to find any statistical difference between severe and non-severe groups [52].
- Cardiovascular disease: Chen et al. found that cardiovascular disease was more frequent in deceased patients in comparison to recovered (14% vs. 4%) [17]. In the study of Zhang et al., cardiovascular disease was observed in 10% of the patients included, with a significant difference between severe and non-severe forms of COVID-19 (23.6% versus 5.4%, *P* < 0.001) [56]. In the study of Zhou et al., coronary artery disease was observed in 8% of the patients included, with a statistically significant difference between non-survivor and survivor (24% versus 1%, *P* < 0.0001) [58] [54]. Kuno et al. found that among 8438 patients with COVID-19, 8.6% had CAD, 8.1% peripheral artery disease, and 6.9% heart failure [33]. Patients with CAD, peripheral artery disease, or heart failure presented higher rates of mechanical ventilation and mortality in all age groups. In the retrospective study of Guo et al., the authors found that 35.3% of patients had a cardiovascular disease in their medical history (coronary heart disease, cardiomyopathy, hypertension) [25]. Mortality rates were 13.33% for patients without cardiac injury and 69.44% for patients with cardiac injury and cardiovascular disease in their medical history. Patients with cardiovascular disease were more likely to have myocardial injury during hospital stay. Coronary heart disease and chronic heart failure were statistically significantly different in patients with cardiac injury in comparison with patients with no cardiac injury in the study of Shi et al. (*P* < 0.001) [43]. Coronary heart disease and chronic heart failure were statistically significantly different in the group of non-survivors in comparison with the survivor group in the study of Shi et al. (*P* < 0.001) [44]. Contrary, Du et al. did not find any statistical difference between ICU and non-ICU groups (*P* = 0.349) [23]. Cardiovascular disease was observed in 15% of the patients included in the study of Huang et al., and no statistical difference was observed between ICU and non-ICU groups (*P* = 0.32) [27]. Chen et al. did not find a statistically significant difference in coronary artery disease between severe and critical forms (*P* = 0.75) [16]. Deng et al. found coronary artery disease in 13.4% of the cases with not finding a statistically significant difference between severe and non-severe forms (*P* = 0.25) [19].
- Cerebrovascular disease: Chen et al. found that cerebrovascular disease was more frequent in deceased patients in comparison to recovered (4% vs.0%) [17]. Cerebrovascular diseases were observed also in 13.5% of the patients in the study of Yang et al.; the incidence was higher in non-survivors in comparison to survivor groups (22 vs. 0%) [53]. Yu et al. found cerebrovascular disease in 6.6% of the patients [54]. In the study of Zhang et al., cerebrovascular disease was observed in 6.8% of the patients included, with a statistically significant difference between severe and non-severe forms of COVID-19 (20% versus 2.4%, *P* < 0.001) [56]. Cerebrovascular disease was statistically significantly different in patients with cardiac injury in comparison with patients with no cardiac injury in the study of Shi et al. (15.9% versus 2.7%, *P* = 0.001) [43]. Cerebrovascular disease and chronic heart failure were statistically significantly different in the group of non-survivors in comparison with the survivor group in the study of Shi et al. (12.9% versus 2.3%, *P* < 0.001) [44].
- Diabetes: Chen et al. found that diabetes was more frequent in deceased patients in comparison to recovered (48% vs.24%) [17]. For Du et al., diabetes was observed in 22% of the patients included [22]. Similarly, Yu et al. found diabetes in 20.8% of the cases, 4.4% with organ damage and 16.4% without organ damage [54]. Xie et al. found that patients with diabetes had a higher prevalence of severe form (*P* = 0.032) [51]. Kunal et al. found diabetes in 32.4% of the patients [32] with a statistically significant difference between non-survivor and survivor (*P* = 0.05) and between patients that developed or not acute cardiac injury during in-hospital stay (*P* = 0.005). Wan et al. found diabetes in 8.9% of patients: the percentage of diabetes was higher between severe and mild forms of COVID-19 (22.5 vs. 3.1%) [50]. Diabetes was observed also in 17% of the patients in the study of Yang et al.: the incidence was higher in non-survivors in comparison to survivor groups (22 vs. 10%) [53]. In the study of Zhou et al., diabetes was observed in 19% of the patients included, with a significant difference between non-survivor and survivor (31% versus 14%, *P* = 0.0051) [58]. Chen et al. found a significant difference in diabetes between severe and critical forms (33.3% versus 80%, *P* < 0.01) [16]. Diabetes was significantly different in patients with cardiac injury in comparison with patients with no cardiac injury in the study of Shi et al. (24.4% versus 12%, *P* = 0.008) [43]. Diabetes was statistically significantly different in the group of non-survivors in comparison with the survivor group in the study of Shi et al. (27.4% versus 13.1%, *P* = 0.004) [44]. Contrariwise, Xiong et al. found that diabetes was high in patients included (16.4%); however, the authors missed to find any statistical difference between severe and non-severe groups [52]. Diabetes was frequent in the multicenter observational study by Du et al. (31.2%); no statistical difference was observed between ICU and non-ICU groups (*P* = 0.386) [23]. Huang et al. found diabetes in 20%; however, the authors missed to find any statistical difference between ICU and non-ICU groups (*P* = 0.16) [27]. Deng et al. found diabetes in 17% of the cases with not finding a statistically significant difference between severe and non-severe forms (*P* = 0.18) [19].
- Chronic respiratory diseases: Chen et al. found that chronic respiratory diseases were more frequent in deceased patients in comparison to recovered (10% vs. 4%) [17]. Chronic respiratory disease was observed in 15.6% of the patients included in the multicenter observational study by Du et al. (31.2%); they observed a higher percentage of chronic respiratory diseases in non-ICU groups reaching a statistically significant difference (*P* = 0.036) [23]. Chronic respiratory diseases were observed also in 8% of the patients in the study of Yang et al.; the incidence was slightly higher in survivors in comparison to non-survivor groups (10 vs. 6%) [53]. Yu et al. found chronic respiratory diseases in 6.6% of the patients [54]. In the study of Zhou et al., chronic obstructive lung disease was observed in 3% of the patients included, with a statistically significant difference between non-survivor and survivor (7% versus 1%, *P* = 0.047) [58]. Arentz et al. found 33.3% of chronic obstructive pulmonary diseases, 28.6% of obstructive sleep apnea, and 9.1% of asthma in patients included [12]. Inversely, Deng et al. found chronic obstructive pulmonary disease in 13.4% of the cases; no statistically significant difference was found between severe and non-severe forms (*P* = 0.53) [19]. Shi et al. did not find a significant difference for chronic obstructive pulmonary disease in the group of non-survivors in comparison with the survivor group (*P* = 1.0) [44].

Due to the few cases included, it is difficult to draw any conclusion to the real impact of malignancy chronic kidney disease, chronic liver disease as risk factors on COVID-19 [17, 22, 23, 25, 27, 32, 44, 52, 54, 56–58, 63]. In one study, chronic kidney failure represented the most common comorbidities among COVID-19 patients (47.6%) [12]. Cancer was statistically significantly different in patients with cardiac injury in comparison with patients with no cardiac injury in the study of Shi et al. [43].

### Secondary outcome: cardiac complications and mortality

**Fig. 3:**
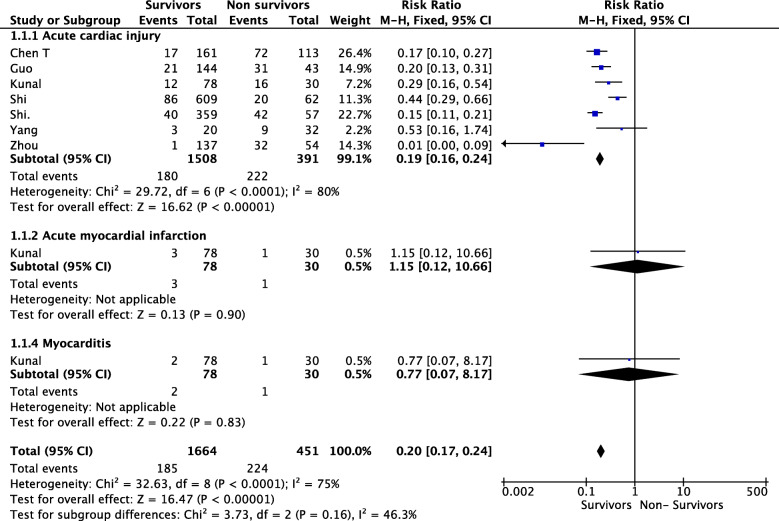
Forest plot for cardiac complications and mortality between survivors and non-survivors. The black diamond at the bottom of the graph represents the average effect size whereas the horizontal line represents the 95% confidence interval (CI). A random-effects model was used. The sample size, the overall effect, 95% CI, and heterogeneity are shown

We included 7 studies investigating 2115 patients in the meta-analysis [17, 25, 32, 43, 44, 53, 58]. The risk ratio (RR) was 0.20 (95% CI: 0.17 to 0.24; *P* < 0.00001, as shown in Fig. 3). Heterogeneity among the studies was significant (*I*^2^ = 0.75). Performing subgroup analysis, for acute cardiac injury patients, we observed a RR of 0.19 (95% CI: 0.16 to 0.24; *P* < 0.00001 as shown in Fig. 3), in favor of survivor. Heterogeneity among the studies was high *I*^2^ = 0.80. For acute myocardial infarction and myocarditis, it was possible to retrieve data only from one study [32].

## Discussion

Emerging evidence showed that, besides acute respiratory disease, cardiac complications may also occur in COVID-19 patients with a severe impact on outcome [8]. Consequently, it is extremely important to further evaluate and share awareness regarding the correlation between COVID-19 and cardiovascular implications.

In our systematic review, we observed that the rate of patients suffering from an acute cardiac injury or myocardial infarction varied among the studies. This can be explained partially by the heterogeneity of the studies included. However, ACI represented the predominant cardiac complications in COVID-19 patients in several studies included (from 20 to 45% of the patients). Patients with acute cardiac injury seemed to be significantly older and with comorbidities. Patients with cardiac injury were more likely to develop complications during the hospital stay with higher mortality rates. ACI was found to be an independent risk factor for the severity of SARS-CoV-2 infection and an independent predictor of mortality. In our meta-analysis, we found a statistically significant difference between non-survivor and survivor in patients with acute cardiac injury (*P* < 0.0001; *I*^2^ = 80%). Myocardial infarction, heart failure, and cardiogenic shock were also described in the literature, with a smaller incidence among COVID-19 patients. Due to the paucity of prospective studies with data mainly basing on case reports, it was not possible to draw any conclusion regarding Takotsubo, myocarditis, and pericardial effusion. Remarkably, the incidence rate of arrhythmia events during hospitalization varied widely among the studies (from 3 to 60%). This can be explained by the wide difference of inclusion criteria between the studies. Several ECG alterations were described (i.e., sinus tachycardia, ventricular tachycardia, premature beat, sinus bradycardia, atrial fibrillation) at different stages of disease. The presence of arrhythmia should be evaluated also in the context of hypoxemia degree and in relation to cardiac, metabolic, and multi-organ deterioration. Analyzing the studies retrieved, it was not possible to draw any assumption regarding the possible association between arrhythmia and comorbidities. Hypertension seemed to represent the most common comorbidities in COVID-19 patients (from 30 to 59.8%); however, the evidence regarding the relation between hypertension and severity of the disease and mortality was inconclusive. The prevalence of cardiovascular disease was high in this group of patients (up to 57%); in particular, coronary artery disease seemed to be around 10% of the cases. In the majority of the studies retrieved, patients with CVD had a higher prevalence of severe form, ICU admission, and higher mortality rates. Furthermore, diabetes emerged as an important risk factor in COVID-19 patients although the evidence is scarce. Due to the lack and the heterogeneity of the cases included, it was not possible to draw conclusions regarding the real role of cerebrovascular disease, chronic respiratory diseases, malignancy, chronic kidney disease, and chronic liver disease as risk factors on COVID-19.

Several physio-pathological mechanisms were proposed to explain the cardiovascular manifestations in COVID-19 patients (i.e., direct and indirect mechanisms): etiology is characterized by a complex interaction between virus, host responses, and underlining cardiac comorbidities [9]. Even if patients with several risk factors (i.e., CVD, diabetes, COPD) may be more susceptible to develop cardiac complications, cardiac abnormalities were also observed in patients without cardiovascular comorbidities.

Cardiac injury can be triggered by a number of direct and indirect mechanisms including viral injury and the interplay with host cells (COVID-19 spike protein (S), ACE2 receptor, host serine protease TMPRSS2, cathepsin B, and cathepsin L) [69]. Preliminary autopsy reports found T-lymphocytic infiltration, CD68+ macrophage infiltration, and viral particles within vascular endothelial cells of the heart in addition to diffuse vascular endothelial cell injury [29]. However, no evidence of viral replication has been found within the cardiomyocytes. Additionally, cytokine storms and systemic inflammatory syndromes and the dysregulation of host immune response may play a role [70], although it needs to be further clarified [71, 72]. Hypoxia related to respiratory failure represents another important role of cardiac injury through the same pathophysiological mechanism leading to type 2 myocardial infarction mechanism [73]. The consequent oxygen supply/demand mismatch is not only due to an inadequate oxygen reserve, but it is also due to the increased demand for oxygen and energy during the cytokine storms, systemic inflammatory syndromes, and dysregulation of host immune response. The loss of the normal balance between the pro- and anti-inflammatory system results in an uncontrolled activation of the inflammatory response, in an immune imbalance (involving both the innate and the adaptive immune response systems), and in a consequent inability of the host to limit the inflammation [74, 75]. IL-6 and catecholamines are responsible for the increase of core body temperature, heart rate, and cardiac oxygen consumption as well. The consequent increase of beat per minutes reduces the filling time with a consequent decrease in myocardial perfusion. Even more, systemic inflammatory response and the consequent release of several inflammatory mediators (i.e., cytokines, chemokines) lead to endothelial dysfunction with coronary artery spasm, thrombosis, and further decrease in heart blood supply. Both hypoxia and pro-inflammatory status lead to mitochondrial dysfunction, alterations of ion channels, and changes of autonomic autoregulation. Alteration of calcium channels and consequent reduction of intracellular calcium led to an impairment of the contractile activity of myocytes [76]. Additionally, reduced oxygen delivery, leading to tissue hypoxia, may have the potential to trigger signaling networks (i.e., hypoxia-triggered signaling pathways) with possible effects in tissue modeling processes and cardiovascular disorders. In particular, hypoxia-inducible factor-1α (HIF-1α) and HIF-2α are transcription factors responsible for the transcriptional cellular responses to hypoxia [77]. Coagulation abnormalities are frequent in COVID-19 patients with several consequences for the cardiovascular system. Depending on the severity of pulmonary embolism, the consequence for the cardiovascular system can range from worsen hypoxemia (with the aforementioned consequences) to right heart failure. Even more, the formation of occlusive thrombus can be observed in the coronary artery itself leading to infarction or inside the cardiac chambers [78]. In addition, electrolyte imbalances are known for their potential dangerous consequence on the cardiovascular system [79]. The renin–angiotensin system plays a central role in controlling fluid and electrolyte balance. This mechanism can partially explain the frequent electrolyte imbalance that has been described in COVID-19 patients. In particular, hyponatremia and hypokalemia are common in COVID-19 patients and may be used as a marker of severity for a rapid screen [80]. Finally, drug-related heart injury and drug interaction were described in COVID-19 patients. In particular, QT-interval prolongation has been observed with the use of hydroxychloroquine, azithromycin, and protease inhibitors [81] and interactions between antiviral agents and some antiarrhythmics and anti-coagulants were described [82]. Another important aspect to analyze is the effects of cardiac complications in COVID-19 patients on mortality. In our meta-analysis, we found a statistically significant difference of acute cardiac complications between non-survivor and survivor groups (*P* < 0.0001). These results have to be weighted on the high heterogeneity between the studies retrieved (*I*^2^ = 80%). However, analyzing the Forrest plot is clear to notice the higher number of cardiovascular events in the non-survivors’ group in comparison to survivors. This aspect is worth to be underlined. Consequently, the cardiovascular complication in COVID-19 patients must be considered a priority during the clinical evaluation of this kind of patients.

We anticipated major limitations of this review: first of all, the heterogeneity in cardiac injury definitions. The authors of this systematic review reported the definition provided by the authors for each article retrieved. It is critical to stress further the importance to follow international consensus definition in order to reach evidence and avoid biases in patients enrollment and result interpretation. In the articles retrieved, acute cardiac injury was defined as an increase in serum levels of troponin T above the 99th percentile *and/or* new abnormalities in ECG/echocardiographic evaluations. In the 2018 “Fourth Universal Definition of Myocardial Infarction,” myocardial injury was defined as “Detection of an elevated cTn value above the 99^th^ percentile URL” [73]. Acute cardiac injury is defined when concomitantly to myocardial injury a *raise and/or fall of cTn values* occurs. In case of a *persistently elevated cTn level, cardiac injury* is defined as chronic.

The authors described acute myocardial infarction as “acute myocardial injury with clinical evidence of acute myocardial ischemia.” Consequently, for the diagnosis of acute myocardial infarction, there is a need to observe, on top of ACI, at least one of the following symptoms of myocardial ischemia: ECG changes, development of Q waves, documentation of coronary thrombus, and TTE abnormalities [73]. However, the widespread use of a homogeneous and standardized definition allows comparison and trend analysis between studies over the time. The importance of a clear and internationally approved definition should be also stressed for myocarditis [83]. Approximately half of the studies retrieved used cardiac magnetic resonance evaluation to reach a diagnosis of myocarditis. However, only three articles referred to specific international guidelines/recommendations for the diagnosis of myocarditis.

Second, the long-term consequences of cardiac manifestations in COVID-19 patients remain basically uncertain due to the limited period of time for post-trial follow-up. A relatively short follow-up period may fail to evaluate the real impact of cardiac complications on outcome. However, follow-up represented a limitation to take into account. Third, we included several case reports and case series. Besides the debatable role of case reports in the era of evidence-based medicine, case reports present several interesting aspects not to be underestimated especially in the COVID-19 pandemic period. Case reports can have an important impact on clinical practice. Case reports can allow a rapid identification of a new disease or complications, consequently can represent an important warning signal, and can promote communication between the medical community. Observation to publication time is short, and this can lead to rapid detection of unusual or harmful clinical findings or complications. As a consequence, case reports can change clinical practice and stimulate the design of further studies. Nevertheless, there is a huge need of well-designed observational studies. Few studies were focused on a specific cardiac complication. Indeed, in order to reach evidence, it is vital to perform large well-designed observational studies aiming to investigate a specific aspect of cardiac complications, following clear and well-standardized definitions.

## Conclusions

Acute cardiac injury represented the prevalent cardiac complications in COVID-19 patients. Patients with acute cardiac injury appeared to be significantly older, with comorbidities, more prone to present complications, and with greater mortality rates. Arrhythmic complications have to be carefully considered by physicians in COVID-19 patients for the possible consequences and complications. Even if inconclusive, it seemed that the presence of coexisting medical conditions is prominent in COVID-19 patients. Few studies were focused on a specific type of cardiac complications. Indeed, in order to reach evidence, it is vital to perform large well-designed observational studies aiming to examine the prevalence of specific cardiac complications, following clear and well-standardized definitions.

## Data Availability

Not applicable

## Abbreviations

COVID-19: Coronavirus disease
ACE inhibitors: Angiotensin-converting-enzyme inhibitors
SARS-CoV-2: Severe acute respiratory syndrome coronavirus 2
PRISMA: The Preferred Reporting Items for Systematic Reviews and Meta-Analyses
MEDLINE: US National Library of Medicine database
CENTRAL: Cochrane Central Register of Controlled clinical studies
RCT: Randomized controlled trial
COPD: Chronic obstructive pulmonary disease
EKG: Electrocardiogram
TTE: Transthoracic echocardiogram
PaO_2_/FiO_2_: Ratio of arterial oxygen partial pressure to fractional inspired oxygen
PaO_2_: Arterial oxygen partial pressure
PaCO_2_: Arterial carbon dioxide partial pressure
GCS: Glasgow Coma Scale
CRRT: Continuous renal replacement therapy
ACI: Acute cardiac injury
CV: Cardiovascular
OR: Odds ratio
CI: Confidence interval
ACEI/ARB: Angiotensin-converting-enzyme inhibitors/angiotensin II receptor blocker
BNP: B-type natriuretic peptide
ARDS: Acute respiratory distress syndrome
RV: Right ventricle
LV: Left ventricle
LVEF: Left ventricular ejection fraction
EF: Ejection fraction
CI: Cardiac index
ESC: European Society of Cardiology
STEMI: ST-elevation myocardial infarction
LVED: Left ventricular end-diastolic pressure
PCI: Percutaneous coronary intervention
LAD: Left anterior descending artery
LVEF: Left ventricular ejection fraction
LCX: Left circumflex artery
RCA: Right coronary artery
MOF: Multi-organ failure
TTS: Takotsubo syndrome
CK-MB: Creatine kinase myocardial band
MRI: Magnetic resonance imaging
CT: Computed tomography
LVEDV: Left ventricular end-diastolic volume
PE: Pleural effusion
PAM: Mean arterial pressure
AF: Atrial fibrillation
VT: Ventricular tachycardia
VF: Ventricular fibrillation
ICU: Intensive care unit
PAPs: Pulmonary artery pressure
GLS: Global longitudinal strain
AT: Pulmonic flow acceleration time velocity
CVD: Cardiovascular disease
CAD: Coronary artery disease
cTn: Cardiac troponin
URL: Upper reference limit
S: Spike protein
HIF1-α: Hypoxia-inducible factor-1α
